# Joint nausea and fatigue profiles during chemotherapy identify patients with a higher symptom burden

**DOI:** 10.1007/s00520-026-10552-x

**Published:** 2026-03-24

**Authors:** Komal P. Singh, Bruce A. Cooper, Kathryn Ruddy, Keenan Pituch, Felipe Batalini, Steven M. Paul, Marilyn Hammer, Yvette P. Conley, Jon D. Levine, Christine Miaskowski

**Affiliations:** 1 Mayo Clinic Cancer Center, Phoenix, AZ USA; 2 Department of Physiological Nursing, School of Nursing, University of California, 490 Illinois Street, Floor 12, San Francisco, CA 94143–0610 USA; 3 Mayo Clinic Cancer Center, Rochester, MN USA; 4 Edson College of Nursing and Health Innovation, Arizona State University, Phoenix, AZ USA; 5 Dana Farber Cancer Institute, Boston, MA USA; 6 School of Nursing, University of Pittsburgh, Pittsburgh, PA USA; 7 School of Medicine, University of California, San Francisco, CA USA

**Keywords:** Cancer, Chemotherapy, Fatigue, Multimorbidity, Nausea

## Abstract

**Purpose:**

Identify subgroups of oncology patients with distinct joint chemotherapy-induced nausea (CIN) AND morning fatigue profiles and distinct joint CIN AND evening fatigue profiles, as well as modifiable and non-modifiable risk factors.

**Methods:**

Oncology patients receiving chemotherapy completed self-report questionnaires that provided information on demographic and clinical characteristics, as well as on CIN and morning and evening fatigue. The three symptoms were assessed six times over two cycles of chemotherapy. Joint latent class profile analyses (LCPA) were performed to identify subgroups of patients with distinct joint symptom profiles. Parametric and non-parametric tests were used to evaluate for differences in modifiable and non-modifiable risk factors among the profiles.

**Results:**

Five and four subgroups were identified with distinct joint CIN and morning fatigue and distinct joint CIN and evening fatigue profiles, respectively. Risk factors associated with membership in the worse profiles included younger age, lower annual household income, high comorbidity burden, lower functional status, self-reported diagnosis of depression, and higher levels of neuropsychological and gastrointestinal symptoms.

**Conclusions:**

Across both LCPAs, 60% of the sample reported CIN with occurrence rates that ranged from approximately 30% to 90%. In addition, wide variations were found in both morning and evening fatigue severity scores depending on the distinct profile. These initial findings suggest that CIN co-occurs with both morning and evening fatigue. The co-occurrence of CIN and fatigue may be related to shared biological mechanisms that warrant evaluation in future studies.

## Introduction

Patients receiving cancer chemotherapy experience an average of 10 to 12 co-occurring symptoms [1]. Two of the most common symptoms reported by these patients are chemotherapy-induced nausea (CIN) and fatigue. Despite advances in antiemetic regimens, occurrence rates for CIN range between 30% and 60% [2]. Similarly, occurrence rates for fatigue range from 52% [3] to 85% [4]. These wide ranges in prevalence rates demonstrate that a large amount of inter-individual variability exists in both symptoms.

While often studied as single symptoms, emerging evidence suggests that variations in fatigue severity are associated with the occurrence of CIN. For example, in a cross-sectional study that used latent class analysis (LCA) [5], three classes of breast cancer patients with distinct symptom profiles were identified (i.e., tired; disrupted sleep and tired; and pain, disrupted sleep, and tired). Except for the tired class, nausea was a highly prevalent symptom in the other two classes. In a longitudinal study of patients with breast cancer [6], higher levels of pre-treatment nausea were a significant risk factor for more severe fatigue following one cycle of chemotherapy. In another study that used latent growth modeling to evaluate for associations between fatigue and other symptoms over two cycles of chemotherapy [7], breast cancer patients in the higher fatigue severity class reported more days with moderate-to-severe nausea. While associations between diurnal variations in fatigue severity and CIN were not evaluated, these findings suggest that the co-occurrence of these two symptoms may have additive or synergistic effects. Of note, risk factors associated with inter-individual variability in the co-occurrence of CIN and fatigue were not reported.

### Inter-individual variability and common and distinct risk factors for CIN and fatigue

Recent work by our research team using hierarchical linear modeling and latent variable modeling characterized inter-individual variability in and risk factors associated with the occurrence of CIN [8–11] and the severity of morning [12, 13] and evening [14, 15] fatigue in oncology patients receiving chemotherapy. For these three symptoms, a comprehensive list of demographic and clinical characteristics, as well as neuropsychological symptoms, was evaluated as potential risk factors for more severe symptoms. In brief, in the LCA for CIN [8], four distinct profiles were identified based on occurrence rates (i.e., None (41%), Increasing–Decreasing (21%), Decreasing (9%), High (29%)).

In terms of fatigue, previous work demonstrated that morning and evening fatigue are distinct symptoms [16] that share common and distinct risk factors [17, 18] and biological mechanisms [19, 20]. For example, using latent profile analysis (LPA), four distinct profiles were identified for morning (i.e., Very Low, Low, High, Very High) [12] and for evening (i.e., Low, Moderate, High, Very High) [15] fatigue. As shown in Table 1, the most common risk factors associated with a higher burden for all three symptoms were younger age, having childcare responsibilities, having a lower functional status and a higher comorbidity burden, as well as a self-reported diagnosis of depression. Equally important, the worst CIN, morning, and evening fatigue profiles were associated with higher severity scores for the majority of the psychoneurological symptoms that were evaluated.

**Table 1:** Summary of common and distinct risk factors and mechanisms for chemotherapy-induced nausea, morning fatigue, and evening fatigue

Risk factors	Chemotherapy-induced nausea			Morning fatigue		Evening fatigue	
	HLM studies^1, 2^	LCA studies^3, 4^		HLM study^5^	LPA study^6^	HLM study^7^	LPA study^8^
Demographic characteristics							
Younger age	■	■			■		■
Being female					■		■
Higher level of education						■	■
Self-reported being white						■	■
Not married or partnered					■		
Living alone					■		
Not employed					■		
Lower annual household income		■					
Higher annual household income					■		■
Having child care responsibilities	■	■				■	■
Clinical characteristics							
Higher body mass index				■	■		
Lack of regular exercise				■	■		
Lower Karnofsky Performance Status score	■	■		■	■		■
Higher number of comorbid conditions					■	■	
Higher SCQ score	■	■			■		■
Diagnosis of anemia or blood disease		■			■		■
Diagnosis of ulcer or stomach disease		■					
Diagnosis of lung disease					■		
Diagnosis of depression		■			■		■
Having a diagnosis of breast cancer							■
Having breast, GI, or GYN cancer						■	
Received only chemotherapy		■					
Did not receive targeted therapy		■					
14-day chemotherapy cycle		■					
Received highly emetogenic chemotherapy	■	■					
Neuropsychological symptoms							
Higher depression scores	■	■		■	■	■	■
Higher state anxiety scores		■		■	■		■
Higher trait anxiety scores	■	■			■		■
Higher sleep disturbance scores	■	■		■	■	■	■
Higher morning fatigue scores	■	■			■		■
Higher evening fatigue scores		■			■		■
Higher worst pain intensity scores		■					
Higher pain interference scores		■					
Lower attentional function scores		■			■		■
Lower morning energy scores	■	■			■		■
Lower evening energy scores		■			■		■
More likely to have cancer and non-cancer pain					■		■
Additional gastrointestinal symptoms (higher occurrence rates)*							
Dry mouth		■					
Feeling bloated	■	■					
Vomiting	■	■					
Diarrhea		■					
Lack of appetite	■	■					
Increased appetite		■					
Abdominal cramps		■					
Difficulty swallowing	■	■					
Mouth sores	■	■					
Weight loss		■					
Weight gain		■					
Constipation	■	■					
Change in way food tastes		■					
Perturbed KEGG pathways							
Pathway name	Chemotherapy-induced nausea^9, 10^		Morning fatigue^11^			Evening fatigue^11^	
Cytokine-cytokine receptor interaction	■		■			■	
Mitogen-activated protein kinase signaling	■						
Nuclear factor κ-B signaling	■		■				
Chemokine signaling	■		■			■	
Intestinal immune network for IgA production	■		■			■	
Peroxisome proliferator-activated receptor	■						
Interleukin 17 producing helper T cell differentiation	■						
Tight junction	■						
Antigen processing and presentation	■		■			■	
Endocytosis	■		■			■	
Regulation of the actin cytoskeleton	■						
Apoptosis	■		■				
Phosphatidylinositol kinase-protein kinase	■					■	
Phagosome			■			■	
Neutrophil extracellular trap formation			■			■	
T-helper cell 17 differentiation							
Nucleotide-binding and oligomerization domain							
Necroptosis			■				
C-type lectin receptor signaling			■				
Mitogen-activated protein kinase			■				
Natural killer cell-mediated toxicity			■			■	
Complement and coagulation cascades						■	
Platelet activation						■	
B-cell receptor signaling							

*Additional gastrointestinal symptoms were not evaluated in the fatigue studies

■ Indicates the presence of the risk factor

While our previous studies demonstrated positive associations between the occurrence of CIN and morning and evening fatigue severity [10, 11], the reciprocal relationship was not evaluated in the LPAs of morning [12] and evening [15] fatigue. However, in unpublished analyses, significant differences were found among the morning fatigue profiles in the occurrence rates for CIN (i.e., Very Low (30.3%), and Low (37.5%) versus High (58.8%), and Very High (66.9%), *p* < 0.001). In terms of the evening fatigue profiles, significant differences were found in the occurrence rates for CIN (i.e., Low (27.4%) versus Moderate (49.8%), High (48.6%) and Very High (53.6%) classes, *p* < 0.001; unpublished data). Given the deleterious effects of both CIN and fatigue, these analyses suggest that additional research is warranted on the co-occurrence of these two symptoms and associated risk factors in patients receiving chemotherapy.

### Theoretical rationale for the co-occurrence of CIN and fatigue

As noted in several reviews, the mechanisms that underlie CIN [21, 22] and fatigue [23–27] are complex. In terms of CIN, early work focused on variations in genes involved in the serotonin receptor, drug metabolism, and/or drug transport pathways. However, none of the candidate genes evaluated were associated with the occurrence and/or severity of CIN [24]. Early mechanistic studies of fatigue evaluated a variety of biomarkers of inflammation and neuroinflammation [23]. For both symptoms, recent work focused on an evaluation of alterations in pathways involved in inflammation [21, 22] and the gut-brain axis [25, 26]. Additional evidence to support the hypothesis that common and distinct mechanisms underlie CIN and fatigue comes from our gene expression studies [20, 28, 29]. As shown in Table 1, several common pathways involved in inflammation (e.g., cytokine-cytokine receptor interaction, chemokine signaling) and alterations in the gut-brain axis (e.g., intestinal immune network for IgA) were perturbed for CIN, as well as for morning and evening fatigue.

Although previous studies provide valuable information on risk factors for CIN and for diurnal variations in fatigue as single symptoms, none of them evaluated for inter-individual variability in and risk factors for CIN AND fatigue in the same latent variable model. Therefore, the purposes of this study, using latent class profile analyses (LCPA), were to identify subgroups of oncology patients with distinct joint CIN AND morning fatigue profiles as well as distinct joint CIN AND evening fatigue profiles. Once the profiles were identified, for each LCPA, differences among the profiles in demographic and clinical characteristics, severity scores for neuropsychological symptoms, and occurrence rates for additional gastrointestinal symptoms were evaluated. The identification of modifiable risk factors for the co-occurrence of CIN AND fatigue will inform tailored interventions, as well as future mechanistic studies.

## Methods

### Patients and settings

As previously described [8], eligible patients were ≥ 18 years; had a diagnosis of breast, gastrointestinal, gynecological, or lung cancer; had received chemotherapy within the preceding four weeks; were scheduled to receive at least two additional cycles of chemotherapy; were able to read, write, and understand English; and gave written informed consent. Patients were recruited from two Comprehensive Cancer Centers, one Veteran’s Affairs hospital, and four community-based oncology programs.

## Study procedures

The study was approved by the Institutional Review Board at each of the study sites. Of the 2234 patients approached, 1338 consented to participate and provided evaluable data on the occurrence of CIN and the severity of morning and evening fatigue for this analysis. Patients’ refusal to participate was related to being overwhelmed with their cancer treatment.

Eligible patients were approached in the infusion unit during their first or second cycle of chemotherapy to discuss participation in the study. After obtaining written informed consent, patients completed measures of CIN and morning and evening fatigue in their homes a total of six times over two cycles of chemotherapy (i.e., prior to chemotherapy administration (Assessments 1 and 4), approximately 1 week after chemotherapy administration (Assessments 2 and 5), and approximately 2 weeks after chemotherapy administration (Assessments 3 and 6)). Additional information used in this analysis were obtained at enrollment (i.e., prior to the second or third cycle of chemotherapy).

## Instruments

### Demographic and clinical characteristics

Patients completed a demographic questionnaire, Karnofsky Performance Status (KPS) scale [30], Self-Administered Comorbidity Questionnaire (SCQ) [31], Alcohol Use Disorders Identification Test [32], and smoking history questionnaire. Medical records were reviewed for disease and treatment information.

## Assessment of CIN occurrence

The nausea item from the Memorial Symptom Assessment Scale (MSAS) was used to assess for the occurrence of CIN at each of the six assessments. The MSAS is a valid and reliable measure that evaluates the occurrence, severity, frequency, and distress of 32 common symptoms [33].

## Assessment of morning and evening fatigue

The 18-item Lee Fatigue Scale (LFS) was designed to assess physical fatigue and energy [34]. Each item was rated on a 0 to 10 numeric rating scale. Mean scores were calculated for the 13 fatigue items and 5 energy items, respectively. Higher scores indicate greater fatigue severity and higher levels of energy. Patients rated each item based on how they felt within 30 min of awakening (i.e., morning fatigue) and prior to going to bed (i.e., evening fatigue). The LFS has an established cutoff score for clinically meaningful levels of fatigue (i.e., ≥3.2 for morning fatigue, ≥5.6 for evening fatigue) [35].

## Assessment of neuropsychological symptoms

An evaluation of other common symptoms was done using valid and reliable instruments. These symptoms and their respective measures were depressive symptoms (Center for Epidemiological Studies-Depression Scale (CES-D) [36]); trait and state anxiety (Spielberger State-Trait Anxiety Inventories (STAI-S and STAI-T) [37]); cognitive function (Attentional Function Index (AFI) [38]); sleep disturbance (General Sleep Disturbance Scale (GSDS) [35]); morning and evening energy (Lee Fatigue Scale (LFS) [34]); and pain (Brief Pain Inventory (BPI) [39]).

## Assessment of additional gastrointestinal symptoms

A modified version of the MSAS was used to evaluate the occurrence of 13 common gastrointestinal symptoms associated with chemotherapy or the cancer itself: dry mouth, feeling bloated, vomiting, diarrhea, lack of appetite, increased appetite, abdominal cramps, difficulty swallowing, mouth sores, weight loss, weight gain, constipation, and change in the way food tastes [33].

## Data analyses

Separate joint LCPAs were done for CIN and morning fatigue and CIN and evening fatigue [40, 41]. These joint LCPAs were done using CIN occurrence rates (yes/no) and fatigue severity scores. Prior to performing the LCPAs, patients who responded “no” to the nausea item on the MSAS for 5 or 6 assessments (i.e., these patients did not experience nausea over the two cycles of chemotherapy; *n* = 543) were identified and their morning and evening fatigue scores were calculated. Then, using Mplus version 8.5 [42], for the remaining patients (*n* = 795), the LCPAs were performed with the combined CIN occurrence rates and fatigue severity scores over the six assessments. This approach provided a profile of these two symptoms.

Estimation was carried out with full information maximum likelihood (FIML) with standard errors and a chi-squared test that are robust to non-normality and non-independence of observations (MLR). Model fit was evaluated to identify the solution that characterized the observed latent class structure with the Bayesian Information Criterion (BIC), entropy, and latent class percents that were large enough to be reliable (i.e., likely to replicate in new samples) [43, 44]. Missing data were accommodated with the use of the Expectation–Maximization (EM) algorithm [45]. This method provides unbiased estimates as long as the missing data process is ignorable (missing at random, missing completely at random, or covariate-dependent missingness). Mixture models such as latent profile (or latent class) analysis are known to produce solutions at local maxima. Therefore, our models were fit from 1000 to 4000 random starts. This approach ensured that the estimated model was replicated many times and not due to a local maximum.

Additional data were analyzed using IBM SPSS Statistics version 29 (IBM Corporation, Armonk, NY). Parametric and nonparametric tests were performed to evaluate for differences among the joint latent classes in demographic and clinical characteristics, neuropsychological symptom scores, and gastrointestinal symptom occurrence rates at enrollment. A *p*-value of <0.05 was considered statistically significant. Post hoc contrasts were done using the Bonferroni procedure.

## Results

### CIN and morning fatigue

#### Latent class analysis

**Fig. 1:**
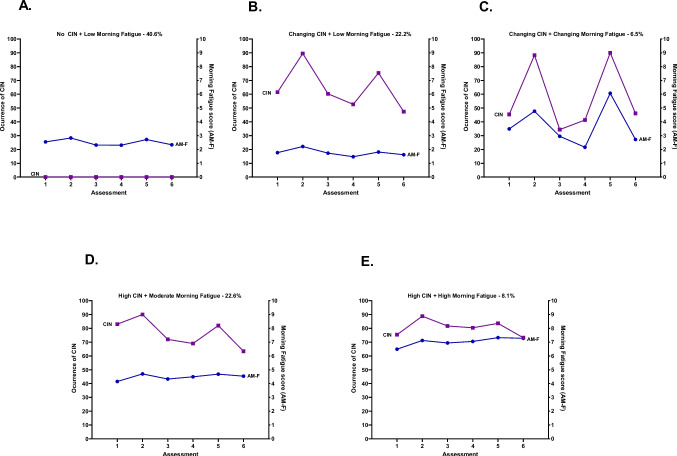
Occurrence of chemotherapy-induced nausea (CIN, left *y*-axis; percentage of patients reporting CIN occurrence) and morning fatigue (AM-F, right *y*-axis; higher scores indicate higher levels of fatigue) scores over two cycles of chemotherapy for subgroups of oncology patients with No CIN and Low Morning Fatigue class (**A**), Changing CIN and Low Morning Fatigue class (B), Changing CIN and Changing Morning Fatigue class (**C**), High CIN and Moderate Morning Fatigue class (**D**), and High CIN and High Morning Fatigue class (**E**)

The 543 patients who had ≤ 1 occurrence of CIN over the six assessments had low morning fatigue scores and were labeled No CIN and Low Morning Fatigue (Both Low-AMF). For the joint CIN and morning fatigue LCPA, a 4-class solution was selected (see details in Table 2). As shown in Fig. 1, 40.6% of the patients were in the No CIN and Low Morning Fatigue class (i.e., Both Low-AMF); 22.2% were in the Changing CIN and Low Morning Fatigue class (i.e., Changing CIN-Low AMF); 6.5% were in the Changing CIN and Changing Morning Fatigue class (i.e., Both Changing-AMF); 22.6% were in the High CIN and Moderate Morning Fatigue class (i.e., High CIN-Moderate AMF); and 8.1% were in the High CIN and High Morning Fatigue class (i.e., Both High-AMF). For the Both Changing-AMF class, the occurrence of CIN and severity of morning fatigue scores increased at assessments 2 and 5 (i.e., the week following the administration of chemotherapy). For the Both Low-AMF and Both High-AMF classes, CIN occurrence and morning fatigue severity scores remained relatively stable over time.

**Table 2:** Latent Profile Solutions and Fit Indices for Nausea Occurrence and Morning and Evening Fatigue

Chemotherapy-induced Nausea and Morning Fatigue					
Model	LL	AIC	BIC	Entropy	VLMR
1 Class	–11134.51	22323.03	22449.34	n/a	n/a
2 Class	–10826.90	21733.80	21920.93	0.77	615.23 ^‡^
3 Class	–10686.99	21479.99	21727.94	0.82	279.81 ^+^
4 Class^a^	–10586.09	21304.18	21612.95	0.81	63.45 ^*^
5 Class	–10516.28	21190.57	21560.16	0.78	ns
Chemotherapy-induced Nausea and Evening Fatigue					
Model	LL	AIC	BIC	Entropy	VLMR
1 Class	–10445.72	20945.44	21071.75	n/a	n/a
2 Class	–10232.61	20545.23	20732.36	0.70	426.21 +
3 Class ^b^	–10114.82	20335.64	20583.59	0.69	235.59 **
4 Class	–10026.44	20184.88	20493.65	0.76	ns

Baseline entropy and VLMR are not applicable for the one-class solution

*p < .05; ^**^p = .001; ^+^*p* = .0001; ^‡^*p* < .00005

^a^The 4-class solution was selected for CIN and morning fatigue because the BIC for that solution was lower than the BIC for the 3-class solution. In addition, the VLMR was significant for the 4-class solution, indicating that four classes fit the data better than three classes. Although the BIC for the 5-class was lower than the BIC for the 4-class solution, the VLMR was not significant for the 5-class solution, indicating that too many classes had been extracted

^b^The 3-class solution was selected for CIN and evening fatigue because the BIC for that solution was lower than the BIC for the 2-class solution. In addition, the VLMR was significant for the 3-class solution, indicating that three classes fit the data better than two classes. Although the BIC for the 4-class was lower than the BIC for the 3-class solution, the VLMR was not significant for the 4-class solution, indicating that too many classes had been extracted

*AIC* Akaike’s Information Criterion; *BIC* Bayesian Information Criterion; *CIN* chemotherapy-induced nausea; *LL* log-likelihood; *n/a* not applicable; *ns* not significant; *VLMR* Vuong-Lo-Mendell-Rubin likelihood ratio test for the K vs. K-1 model

#### Demographic and clinical characteristics

Differences among the five classes are listed in Table 3. In brief, compared to the Both Low-AMF class, the other four classes were younger. Compared to the Both Low-AMF and Changing CIN-Low AMF classes, the other three classes had lower KPS scores, were more likely to self-report a diagnosis of depression, and had received a neurokinin-1 receptor antagonist and two other antiemetics. Compared to the other four classes, the Both High-AMF class had a lower annual income, was less likely to exercise on a regular basis, and had higher SCQ scores.

**Table 3:** Differences in Demographic and Clinical Characteristics Among the CIN and Morning Fatigue Latent Profiles at Enrollment

Characteristic	No CIN and Low Morning Fatigue (0)	Changing CIN and Low Morning Fatigue (1)	Changing CIN and Changing Morning Fatigue (2)	High CIN and Moderate Morning Fatigue (3)	High CIN and High Morning Fatigue (4)	Statistics
	40.6% (*n*=543)	22.2% (*n*=297)	6.5% (*n*=87)	22.6% (*n*=302)	8.1% (*n*=109)	
	Mean (SD)	Mean (SD)	Mean (SD)	Mean (SD)	Mean (SD)	
Age (years)	60.0 (12.1)	57.0 (12.1)	52.7 (13.7)	54.7 (11.8)	54.0 (11.5)	F = 15.19, *p* <.001 0 > 1, 2, 3, and 4 1 > 2
Education (years)	16.3 (3.1)	16.4 (3.1)	16.2 (3.0)	16.0 (2.9)	15.7 (2.9)	F = 1.45, *p* = .215
Body mass index (kg/m^2^)	26.2 (5.4)	25.6 (5.1)	26.7 (5.4)	26.1 (5.9)	27.7 (7.2)	F = 3.14, *p* = .014 1 < 4
Alcohol Use Disorders Identification Test score	3.1 (2.3)	2.9 (2.3)	2.9 (2.5)	2.8 (2.8)	3.0 (2.8)	F = 0.34, *p* = .855
Karnofsky Performance Status score (KPS)	83.1 (11.8)	82.8 (11.8)	78.3 (13.0)	75.4 (11.3)	70.7 (12.0)	F = 40.39, *p* <.001 0 and 1 > 2, 3 and 4; 2 and 3 > 4
Number of comorbid conditions	2.4 (1.4)	2.2 (1.3)	2.3 (1.3)	2.5 (1.5)	3.1 (1.8)	F = 8.55, *p* <.001 1 < 3 and 4 2 < 4; 0 < 4
Self-administered Comorbidity Questionnaire score	5.2 (3.0)	4.9 (2.7)	5.4 (2.9)	5.8 (3.2)	7.5 (4.5)	F = 16.14, *p* <.001 0, 1, 2 and 3 < 4; 1 < 3
Time since diagnosis (years)	2.2 (4.3)	1.8 (3.7)	1.5 (2.9)	2.0 (3.9)	1.4 (2.7)	KW = 5.50, *p* = .240
Time since diagnosis (years, median)	0.44	0.41	0.37	0.42	0.42	
Number of prior cancer treatments	1.7 (1.6)	1.5 (1.4)	1.3 (1.3)	1.7 (1.5)	1.8 (1.5)	F = 2.55, *p* = .037 No significant pw contrasts
Number of metastatic sites including lymph node involvement^a^	1.3 (1.3)	1.2 (1.1)	1.1 (1.2)	1.2 (1.3)	1.2 (1.2)	F = 1.29, *p* = .274
Number of metastatic sites excluding lymph node involvement	0.9 (1.1)	0.7 (1.0)	0.6 (1.0)	0.8 (1.1)	0.8 (1.0)	F = 2.52, *p* = .040 No significant pw contrasts
MAX2 score	0.16 (0.09)	0.17 (0.07)	0.21 (0.08)	0.18 (0.08)	0.19 (0.08)	F = 5.99, *p* <.001 0, 1 and 3 < 2;
	% (*n*)	% (*n*)	% (*n*)	% (*n*)	% (*n*)	
Gender (% female)	72.9 (395)	73.7 (219)	88.5 (77)	84.8 (256)	85.3 (93)	Χ^2^ = 28.26, *p* <.001 0 and 1 < 2 and 3
Self-reported ethnicity						Χ^2^ = 19.26, *p* = .083
White	70.5 (378)	70.7 (208)	67.4 (58)	69.5 (207)	64.5 (69)	
Asian or Pacific Islander	11.9 (64)	13.3 (39)	14.0 (12)	13.8 (41)	8.4 (9)	
Black	8.4 (45)	7.8 (23)	7.0 (6)	4.4 (13)	7.5 (8)	
Hispanic, Mixed, or Other	9.1 (49)	8.2 (24)	11.6 (10)	12.4 (37)	19.6 (21)	
Married or partnered (% yes)	67.2 (359)	68.7 (202)	66.3 (57)	59.1 (176)	52.3 (56)	Χ^2^ = 14.88, *p* = .005 0 and 1 > 4
Lives alone (% yes)	19.5 (104)	17.7 (52)	17.6 (15)	24.7 (74)	36.1 (39)	Χ^2^ = 20.10, *p* <.001 0, 1, and 2 < 4
Currently employed (% yes)	36.4 (196)	39.7 (116)	32.6 (28)	33.1 (99)	23.9 (26)	Χ^2^ = 9.98, *p* = .041 1 > 4
Annual household income						KW = 36.15, *p* <.001 0, 1, 2 and 3 > 4 0 and 1 > 3
Less than $30,000^+^	13.8 (66)	13.8 (36)	16.7 (14)	23.1 (63)	40.6 (41)	
$30,000 to $70,000	20.9 (100)	20.7 (54)	22.6 (19)	21.6 (59)	19.8 (20)	
$70,000 to $100,000	19.0 (91)	15.3 (40)	19.0 (16)	16.5 (45)	10.9 (11)	
Greater than $100,000	46.3 (222)	50.2 (131)	41.7 (35)	38.8 (106)	28.7 (29)	
Child care responsibilities (% yes)	18.0 (96)	18.8 (54)	26.7 (23)	29.4 (87)	28.3 (30)	Χ^2^ =19.60, *p* <.001 0 and 1 < 3
Elder care responsibilities (% yes)	7.5 (37)	7.5 (20)	2.5 (2)	9.8 (27)	10.1 (10)	Χ^2^ = 5.48, *p* = .242
Past or current history of smoking (% yes)	37.6 (200)	30.8 (91)	34.5 (29)	33.3 (99)	42.6 (46)	Χ^2^ = 6.82, *p* = .146
Exercise on a regular basis (% yes)	70.5 (378)	78.5 (227)	72.9 (62)	69.4 (206)	52.5 (53)	Χ^2^ = 25.30, *p* <.001 0, 1, 2 and 3 > 4
Specific comorbid conditions (% yes)						
Heart disease	7.0 (38)	3.4 (10)	3.4 (3)	6.0 (18)	7.3 (8)	Χ^2^ = 6.05, *p* = .195
High blood pressure	32.8 (178)	29.6 (88)	31.0 (27)	27.8 (84)	25.7 (28)	Χ^2^ = 3.65, *p* = .455
Lung disease	12.9 (70)	9.8 (29)	6.9 (6)	8.9 (27)	17.4 (19)	Χ^2^ = 9.53, *p* = .049 No significant pw contrasts
Diabetes	7.9 (43)	8.1 (24)	8.0 (7)	11.3 (34)	11.9 (13)	Χ^2^ = 4.18, *p* = .383
Ulcer or stomach disease	3.5 (19)	4.4 (13)	5.7 (5)	6.6 (20)	7.3 (8)	Χ^2^ = 5.95, *p* = .203
Kidney disease	0.6 (3)	1.3 (4)	4.6 (4)	1.3 (4)	3.7 (4)	Χ^2^ = 13.17, *p* = .010 0 < 2 and 4
Liver disease	6.6 (36)	7.1 (21)	4.6 (4)	6.3 (19)	5.5 (6)	Χ^2^ = 0.89, *p* = .926
Anemia or blood disease	9.2 (50)	12.5 (37)	17.2 (15)	13.2 (40)	20.2 (22)	Χ^2^ = 13.36, *p* = .010 0 < 4
Depression	14.2 (77)	8.1 (24)	27.6 (24)	26.5 (80)	47.7 (52)	Χ^2^ = 103.84, *p* <.001 0 and 1 < 2, 3 and 4 2 and 3 < 4
Osteoarthritis	13.8 (75)	10.4 (31)	6.9 (6)	11.6 (35)	14.7 (16)	Χ^2^ = 5.20, *p* = .267
Back pain	23.6 (128)	21.2 (63)	19.5 (17)	30.1 (91)	45 (41.3)	Χ^2^ = 23.11, *p* <.001 0, 1 and 2 < 4
Rheumatoid arthritis	4.2 (23)	1.7 (5)	0.0 (0)	2.3 (7)	7.3 (8)	Χ^2^ = 13.69, *p* = .008 1 < 4
Cancer diagnosis						Χ^2^ = 25.74, *p* = .012
Breast cancer	39.4 (214)	36.4 (108)	52.9 (46)	42.7 (129)	38.5 (42)	NS
Gastrointestinal cancer	30.9 (168)	37.4 (111)	14.9 (13)	29.1 (88)	26.6 (29)	0 and 1 > 2
Gynecological cancer	16.9 (92)	14.1 (42)	24.1 (21)	18.5 (56)	22 (20.2)	NS
Lung cancer	12.7 (69)	12.1 (36)	8.0 (7)	9.6 (29)	14.7 (16)	NS
Prior cancer treatment						Χ^2^ = 15.98, *p* = .192
No prior treatment	24.4 (128)	28.6 (82)	31.4 (27)	22.1 (66)	21.0 (22)	
Only surgery, CTX, or RT	41.3 (217)	39.7 (114)	46.5 (40)	44.3 (132)	41.9 (44)	
Surgery and CTX, or surgery and RT, or CTX and RT	21.1 (111)	20.9 (60)	15.1 (13)	18.8 (56)	17.1 (18)	
Surgery and CTX and RT	13.1 (69)	10.8 (31)	7.0 (6)	14.8 (44)	20.0 (21)	
Metastatic sites						Χ^2^ = 20.84, *p* = .053
No metastasis	31.7 (169)	29.9 (87)	40.2 (35)	34.3 (103)	30.3 (33)	
Only lymph node metastasis	17.8 (95)	26.5 (77)	23.0 (20)	22.0 (66)	30.3 (33)	
Only metastatic disease in other sites	23.3 (124)	21.6 (63)	12.6 (11)	20.7 (62)	17.4 (19)	
Metastatic disease in lymph nodes and other sites	27.2 (145)	22.0 (64)	24.1 (21)	23.0 (69)	22.0 (24)	
Receipt of targeted therapy (%yes)	34.6 (182)	29.3 (85)	28.7 (25)	23.7 (71)	26.6 (29)	Χ^2^ = 11.62, *p* = .020 0 > 3
CTX regimen						Χ^2^ = 21.09, *p* = .007
Only CTX	65.4 (344)	70.7 (205)	71.3 (62)	76.3 (228)	73.4 (80)	0 < 3
Only targeted therapy	4.9 (26)	1.0 (3)	1.1 (1)	2.3 (7)	1.8 (2)	0 > 1
Both CTX and targeted therapy	29.7 (156)	28.3 (82)	27.6 (24)	21.4 (64)	24.8 (27)	NS
Cycle length						KW = 11.15, *p* = .025 No significant pw contrasts
14 day cycle	37.8 (204)	48.1 (142)	35.6 (31)	47.1 (140)	38.3 (41)	
21 day cycle	53.5 (289)	44.4 (131)	62.1 (54)	45.8 (136)	57.0 (61)	
28 day cycle	8.7 (47)	7.5 (22)	2.3 (2)	7.1 (21)	4.7 (5)	
Emetogenicity of the CTX regimen						KW = 24.00, *p* <.001 0 < 1, 2 and 3
Minimal/low	24.4 (132)	14.2 (42)	12.6 (11)	19.5 (58)	15.0 (16)	
Moderate	61.0 (330)	66.4 (196)	59.8 (52)	55.6 (165)	62.6 (67)	
High	14.6 (79)	19.3 (57)	27.6 (24)	24.9 (74)	22.4 (24)	
Antiemetic regimen						Χ^2^ = 56.50, *p* <.001
None	10.1 (53)	4.1 (12)	1.1 (1)	8.0 (23)	2.9 (3)	0 > 1
Steroid alone or serotonin receptor antagonist alone	23.4 (123)	18.5 (54)	23.0 (20)	18.5 (53)	14.4 (15)	NS
Serotonin receptor antagonist and steroid	46.8 (246)	57.5 (168)	40.2 (35)	43.2 (124)	43.3 (45)	1 > 0, 2 and 3
NK-1 receptor antagonist and two other antiemetics	19.8 (104)	19.9 (58)	35.6 (31)	30.3 (87)	39.4 (41)	0 and 1 < 2, 3 and 4

^a^Total number of metastatic sites evaluated was 9

^+^Reference group

*CIN* Chemotherapy-induced nausea; *CTX* chemotherapy; *kg* kilograms; *KW* Kruskal Wallis; *m*^*2*^ meters squared; *pw* pairwise; *NK-1* neurokinin-1; *NS* not significant; *RT* radiation therapy; *SD* standard deviation

#### Neuropsychological symptom scores

Compared to the Both Low-AMF and the Changing CIN-Low AMF classes, the other three classes had higher levels of depression, sleep disturbance, and morning and evening fatigue (Table 4). Compared to the Both Low-AMF, the Changing CIN-Low AMF and the Both Changing-AMF classes, the other two classes reported higher levels of trait and state anxiety and lower levels of attentional function. Compared to the Both Low-AMF and the Changing CIN-Low AMF classes, the High CIN-Moderate AMF and the Both High-AMF classes reported higher occurrence rates for both cancer and non-cancer pain. Compared to the other four classes, the Both High-AMF had higher worst pain intensity scores.

**Table 4:** Differences in Neuropsychological Symptom Severity Scores Among the CIN and Morning Fatigue Latent Profiles at Enrollment

Neuropsychological Symptom Scores*	No CIN and Low Morning Fatigue	Changing CIN and Low Morning Fatigue	Changing CIN and Changing Morning Fatigue	High CIN and Moderate Morning Fatigue	High CIN and High Morning Fatigue	Statistics
	(0)	(1)	(2)	(3)	(4)	
	40.6% (*n*=543)	22.2% (*n*=297)	6.5% (*n*=87)	22.6% (*n*=302)	8.1% (*n*=109)	
	Mean (SD)	Mean (SD)	Mean (SD)	Mean (SD)	Mean (SD)	
Center for Epidemiological Studies Depression Scale (≥16.0)	10.3 (8.5)	8.8 (6.1)	14.7 (9.5)	16.7 (9.2)	24.6 (11.7)	F = 92.27, *p* <.001 0 and 1 < 2, 3 and 4; 2 and 3 < 4
Trait Anxiety Inventory (≥32.2)	32.9 (9.7)	31.3 (7.8)	35.7 (9.8)	39.2 (10.3)	45.6 (11.2)	F = 62.69, *p* < .001 0, 1 and 2 < 3 and 4; 1 < 2; 3 < 4
State Anxiety Inventory (≥ 31.8)	31.2 (11.2)	30.2 (10.2)	33.3 (11.1)	37.8 (11.8)	47.3 (14.2)	F = 60.73, *p* < .001 0, 1 and 2 < 3 and 4; 3 < 4
Attentional Function Index (≤ 5 low, 5–7.5 moderate, > 7.5 high)	6.8 (1.7)	7.1 (1.6)	6.3 (1.8)	5.6 (1.4)	4.6 (1.9)	F = 65.71, *p* < .001 0, 1 and 2 > 3 and 4; 0 < 1; 1 > 2; 3 > 4
General Sleep Disturbance Scale (≥ 43.0)	46.7 (19.5)	43.9 (15.7)	56.0 (18.3)	62.3 (16.8)	74.9 (16.70)	F = 94.58, *p* <.001 0 and 1 < 2, 3 and 4; 2 < 3 and 4; 3 < 4
Morning fatigue (≥ 3.2)	2.5 (2.1)	1.8 (1.4)	3.6 (2.3)	4.2 (1.6)	6.6 (1.8)	F = 170.82, *p* < .001 0 and 1 < 2, 3 and 4 0 > 1; 2 and 3 < 4
Evening fatigue (≥ 5.6)	4.8 (2.2)	4.8 (2.1)	5.8 (2.3)	5.9 (1.6)	7.6 (1.5)	F = 54.30, *p* <.001 0 and 1 < 2 ,3, and 4; 2 and 3 < 4
Morning energy (≤ 6.2)	4.6 (2.3)	4.7 (2.4)	4.2 (2.1)	4.1 (1.7)	3.1 (2.3)	F = 13.45, *p* <.001 0 and 1 > 3 and 4; 2 and 3 > 4
Evening energy (≤ 3.5)	3.8 (2.0)	3.5 (2.0)	3.4 (2.2)	3.6 (1.9)	2.3 (1.8)	F = 11.87, *p* < .001 0,1, 2 and 3 > 4
Type of pain (% (*n*))						Χ^2^ = 82.95, *p* < .001
No pain	33.8 (181)	30.1 (88)	21.2 (18)	20.4 (60)	11.3 (12)	0 > 3 and 4, 1 > 4;
Only non-cancer pain	18.1 (97)	18.8 (55)	5.9 (5)	13.6 (40)	11.3 (12)	0 and 1 > 2
Only cancer pain	22.0 (118)	29.5 (86)	40.0 (34)	28.2 (83)	23.6 (25)	0 < 2
Both cancer and non-cancer pain	26.1 (140)	21.6 (63)	32.9 (28)	37.8 (111)	53.8 (57)	0 and 1 < 3 < 4
Worst pain intensity	5.8 (2.5)	5.3 (2.4)	6.5 (2.4)	6.2 (2.5)	7.8 (2.3)	F = 15.81, *p* <.001 0, 1, 2, and 3 < 4; 1 < 2 and 3
Number of pain locations (out of 45)	26.6 (22.1)	29.2 (21.5)	32.1 (20.5)	33.4 (19.7)	35.9 (18.1)	F = 7.91 *p* <.001 0 < 3 and 4; 1 < 4
Pain interference	2.6 (2.4)	2.3 (2.0)	3.2 (2.5)	3.8 (2.4)	5.2 (2.7)	F = 31.92, *p* <.001 0 and 1 < 3 and 4; 2 and 3 < 4

*Numbers in parentheses represent clinically meaningful cutpoint scores for the symptom measures

*CIN* chemotherapy-induced nausea; *SD* standard deviation

#### Gastrointestinal symptom occurrence rates

Compared to the Both Low-AMF and the Changing CIN-Low AMF classes, the High CIN-Moderate AMF and the Both High-AMF classes reported higher occurrence rates for dry mouth, feeling bloated, difficulty swallowing, and change in the way food tastes (Table 5). Compared to the Both Low-AMF class, the other four classes reported higher occurrence rates for vomiting. Compared to the Both Low-AMF class, the Changing CIN-Low AMF, High CIN-Moderate AMF, and the Both High-AMF classes reported higher occurrence rates for diarrhea and constipation.

**Table 5:** Differences in the occurrence of gastrointestinal symptoms among the CIN and morning fatigue latent profiles at enrollment

Occurrence of symptoms	No CIN and Low Morning Fatigue (0) 40.6% (*n* = 543)	Changing CIN and Low Morning Fatigue (1) 22.2% (*n* = 297)	Changing CIN and Changing Morning Fatigue (2) 6.5% (*n* = 87)	High CIN and Moderate Morning Fatigue (3) 22.6% (*n* = 302)	High CIN and High Morning Fatigue (4) 8.1% (*n* = 109)	Statistics
	% (*n*)	% (*n*)	% (*n*)	% (*n*)	% (*n*)	
Dry mouth	38.6 (209)	42.2 (125)	44.8 (39)	55.6 (165)	60.7 (65)	*Χ*^2^ = 33.97, *p* <.001 0 and 1 < 3 and 4
Feeling bloated	23.8 (129)	29.4 (87)	33.3 (29)	45.8 (136)	55.1 (59)	*Χ*^2^ = 68.07, *p* <.001 0 and 1 < 3 and 4; 2 < 4
Vomiting	3.5 (19)	13.2 (39)	18.4 (16)	22.2 (66)	22.4 (24)	*Χ*^2^ = 79.33, *p* <.001 0 < 1, 2, 3 and 4; 1 < 3
Diarrhea	21.4 (116)	32.1 (95)	34.5 (30)	36 (107)	42.1 (45)	*Χ*^2^ = 33.23, *p* <.001 0 < 1, 3 and 4
Lack of appetite	28.8 (156)	39.5 (117)	34.5 (30)	60.6 (180)	61.7 (66)	*Χ*^2^ = 101.07, *p* <.001 0, 1 and 2 < 3 and 4; 0 < 1
Increased appetite	22.1 (120)	24.0 (71)	34.5 (30)	30.3 (90)	30.8 (33)	*Χ*^2^ = 12.26, *p* =.016 No significant pw contrasts
Abdominal cramps	15.9 (86)	20.3 (60)	19.5 (17)	30.6 (91)	42.1 (45)	*Χ*^2^ = 49.71, *p* <.001 0, 1 and 2 < 4; 0 and 1 < 3
Difficulty swallowing	8.3 (45)	11.5 (34)	9.2 (8)	20.2 (60)	33.6 (36)	*Χ*^2^ = 62.42, *p* <.001 0 and 1 < 3 and 4; 2 < 4
Mouth sores	17.0 (92)	17.6 (52)	19.5 (17)	30.0 (89)	26.2 (28)	*Χ*^2^ = 23.69, *p* <.001 0 and 1 < 3
Weight loss	20.1 (109)	26.0 (77)	17.2 (15)	31.3 (93)	38.3 (41)	*Χ*^2^ = 26.13, *p* <.001 0 and 2 < 4; 0 < 3
Weight gain	24.0 (130)	22.3 (66)	32.2 (28)	25.9 (77)	33.6 (36)	*Χ*^2^ = 8.08, *p* =.089
Constipation	33.8 (183)	44.3 (131)	41.4 (36)	55.9 (166)	57.9 (62)	*Χ*^2^ = 48.78, *p* <.001 0 < 1, 3 and 4; 1 < 3 and 4
Change in way food tastes	39.5 (214)	48.3 (143)	52.9 (46)	61.6 (183)	65.4 (70)	*Χ*^2^ = 50.60, *p* <.001 0 and 1 < 3 and 4

*CIN* chemotherapy-induced nausea, *pw* pairwise

### CIN and evening fatigue

#### Latent class analysis

**Fig. 2:**
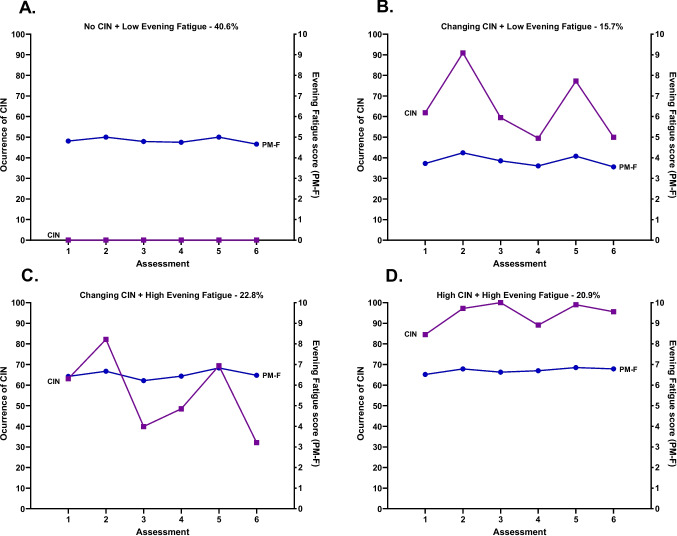
Occurrence of chemotherapy-induced nausea (CIN, left *y*-axis; percentage of patients reporting CIN occurrence) and evening fatigue (PM-F, right *y*-axis; higher scores indicate higher levels of fatigue) scores over two cycles of chemotherapy for subgroups of oncology patients with No CIN and Low Evening Fatigue class (**A**), Changing CIN and Low Evening Fatigue class (**B**), Changing CIN and High Evening Fatigue class (**C**), and High CIN and High Evening Fatigue class (**D**)

The 543 patients who had ≤ 1 occurrence of CIN over the six assessments had low evening fatigue scores and were labeled No CIN and Low Evening Fatigue (Both Low-PMF). For the joint CIN-evening fatigue LCPA, a 3-class solution was selected (see details on Table 2). As shown in Fig. 2, 40.6% of the patients were in the No CIN and Low Evening Fatigue class (i.e., Both Low-PMF); 15.7% were in the Changing CIN and Low Evening Fatigue class (i.e., Changing CIN-Low PMF); 22.8% were in the Changing CIN and High Evening Fatigue class (i.e., Changing CIN-High PMF); and 20.9% were in the High CIN and High Evening Fatigue class (i.e., Both High-PMF). For the Changing CIN-Low PMF class and Changing CIN-High PMF class, the occurrence of CIN increased at assessments 2 and 5, while severity of evening fatigue scores remained stable over time. For the Both Low-PMF and Both High-PMF classes, CIN occurrence rates and evening fatigue severity scores remained relatively stable over time.

#### Demographic and clinical characteristics

Compared to the Both Low-PMF class, the other three classes were younger and were more likely to receive highly emetogenic chemotherapy (Table 6). Compared to the Both Low-PMF and Changing CIN-Low PMF classes, the other two classes were more likely to have childcare responsibilities and lower KPS scores. Compared to the Both Low-PMF and the Changing CIN-Low PMF classes, the Both High-PMF class was more likely to have a higher number of comorbid conditions and a higher SCQ score. Compared to the Both Low-PMF class, the Changing CIN-Low PMF and Both High-PMF classes were likely to have a lower annual household income and receive only chemotherapy.

**Table 6:** Differences in Demographic and Clinical Characteristics among the CIN and Evening Fatigue Latent Profiles at Enrollment

Characteristic	No CIN and Low Evening Fatigue	Changing CIN and Low Evening Fatigue	Changing CIN and High Evening Fatigue	High CIN and High Evening Fatigue	Statistics
	(0)	(1)	(2)	(3)	
	40.6% (*n*=543)	15.7% (*n*=210)	22.9% (*n*=306)	20.9% (*n*=279)	
	Mean (SD)	Mean (SD)	Mean (SD)	Mean (SD)	
Age (years)	60.0 (12.1)	57.0 (12.8)	54.8 (12.0)	54.5 (11.8)	F = 18.22, *p* < .001 0 > 1, 2 and 3
Education (years)	16.3 (3.1)	16.0 (3.0)	16.3 (2.8)	16.1 (3.1)	F = 0.54, *p* = .655
Body mass index (kg/m^2^)	26.2 (5.4)	26.0 (5.3)	26.1 (5.8)	26.5 (6.2)	F = 0.38, *p* = .769
Alcohol Use Disorders Identification Test score	3.1 (2.3)	2.8 (2.4)	3.0 (2.7)	2.8 (2.6)	F = 0.70, *p* = .553
Karnofsky Performance Status score (KPS)	83.1 (11.8)	81.3 (12.9)	78.3 (11.9)	74.8 (12.1)	F = 31.06, *p* < .001 0 and 1 > 2 and 3; 2 > 3
Number of comorbid conditions	2.3 (1.4)	2.3 (1.4)	2.4 (1.4)	2.6 (1.5)	F = 3.35, *p* = .018 0 and 1 < 3
Self-administered Comorbidity Questionnaire score	5.2 (3.0)	5.1 (3.0)	5.6 (3.2)	6.1 (3.6)	F = 6.13, *p* < v.001 0 and 1 < 3
Time since diagnosis (years)	2.2 (4.3)	1.8 (3.9)	1.8 (3.2)	1.8 (3.7)	KW = 2.52, *p* = .472
Time since diagnosis (years, median)	0.44	0.43	0.42	0.39	
Number of prior cancer treatments	1.7 (1.6)	1.5 (1.4)	1.6 (1.5)	1.6 (1.5)	F = 0.95, *p* = .416
Number of metastatic sites including lymph node involvement^a^	1.3 (1.3)	1.2 (1.1)	1.2 (1.2)	1.2 (1.3)	F = 1.41, *p* = .239
Number of metastatic sites excluding lymph node involvement	0.9 (1.1)	0.7 (1.0)	0.7 (1.0)	0.7 (1.0)	F = 2.88, *p* = .035 No significant pairwise contrasts
MAX2 score	0.16 (0.09)	0.18 (0.08)	0.19 (0.08)	0.17 (0.07)	F = 6.38, *p* < .001 0 < 2; 2 > 3
	% (*n*)	% (*n*)	% (*n*)	% (*n*)	
Gender (% female)	72.9 (395)	77.1 (162)	85.0 (260)	79.9 (223)	Χ^2^ = 17.48, *p* < .001 0 < 2
Self-reported ethnicity					Χ^2^ = 19.47, *p* = .021
White	70.5 (378)	61.1 (127)	75.4 (227)	68.1 (188)	1 < 2
Asian or Pacific Islander	11.9 (64)	17.8 (37)	10.3 (31)	12.0 (33)	NS
Black	8.4 (45)	8.7 (18)	5.3 (16)	5.8 (16)	NS
Hispanic, Mixed, or Other	9.1 (49)	12.5 (26)	9.0 (27)	14.1 (39)	NS
Married or partnered (% yes)	67.2 (359)	63.1 (130)	64.7 (196)	59.8 (165)	Χ^2^ = 4.59, *p* = .204
Lives alone (% yes)	19.5 (104)	20.4 (42)	22.0 (67)	25.7 (71)	Χ^2^ = 4.42, *p* = .220
Currently employed (% yes)	36.4 (196)	32.5 (67)	37.7 (114)	31.7 (88)	Χ^2^ = 3.40, *p* = .335
Annual household income					KW = 13.41, *p* = .004 0 > 1 and 3
Less than $30,000^+^	13.8 (66)	20.9 (38)	17.2 (48)	26.4 (68)	
$30,000 to $70,000	20.9 (100)	26.4 (48)	19.4 (54)	19.4 (50)	
$70,000 to $100,000	19.0 (91)	17.6 (32)	17.9 (50)	11.6 (30)	
Greater than $100,000	46.3 (222)	35.2 (64)	45.5 (127)	42.6 (110)	
Child care responsibilities (% yes)	18.0 (96)	15.8 (32)	27.4 (82)	29.2 (80)	Χ^2^ = 22.81, *p* <.001 0 and 1 < 2 and 3
Elder care responsibilities (% yes)	7.5 (37)	8.9 (17)	5.7 (16)	10.4 (26)	Χ^2^ = 4.33, *p* = .228
Past or current history of smoking (% yes)	37.6 (200)	26.7 (55)	33.8 (102)	39.1 (108)	Χ^2^ = 9.97, *p* = .019 0 and 3 > 1
Exercise on a regular basis (% yes)	70.5 (378)	73.9 (150)	75.5 (225)	63.8 (173)	Χ^2^ = 10.50, *p* = .015 2 > 3
Heart disease	7.0 (38)	11.0 (5.2)	3.3 (10)	6.5 (18)	Χ^2^ = 5.39, *p* = .145
High blood pressure	32.8 (178)	30.5 (64)	27.1 (83)	28.7 (80)	Χ^2^ = 3.40, *p* = .334
Lung disease	12.9 (70)	7.6 (16)	9.8 (30)	12.5 (35)	Χ^2^ = 5.33, *p* = .149
Diabetes	7.9 (43)	10.0 (21)	8.5 (26)	11.1 (31)	Χ^2^ = 2.63, *p* = .452
Ulcer or stomach disease	3.5 (19)	5.7 (12)	4.9 (15)	6.8 (19)	Χ^2^ = 4.80, *p* = .187
Kidney disease	0.6 (3)	1.4 (3)	2.3 (7)	2.2 (6)	Χ^2^ = 5.63, *p* = .131
Liver disease	6.6 (36)	5.7 (12)	5.2 (16)	7.9 (22)	Χ^2^ = 1.93, *p* = .587
Anemia or blood disease	9.2 (50)	11.9 (25)	15.4 (47)	15.1 (42)	Χ^2^ = 9.49, *p* = .023 0 < 2
Depression	14.2 (77)	16.2 (34)	22.5 (69)	27.6 (77)	Χ^2^ = 24.94, *p* < .001 0 < 2 and 3; 1 < 3
Osteoarthritis	13.8 (75)	10.5 (22)	11.1 (34)	11.5 (32)	Χ^2^ = 2.38, *p* = .497
Back pain	23.6 (128)	22.9 (48)	25.8 (79)	31.9 (89)	Χ^2^ = 7.79, *p* = .051
Rheumatoid arthritis	4.2 (23)	1.9 (4)	2.0 (6)	3.6 (10)	Χ^2^ = 4.65, *p* = .199
Cancer diagnosis					Χ^2^ = 20.01, *p* = .018
Breast cancer	39.4 (214)	34.3 (72)	45.8 (140)	40.5 (113)	NS
Gastrointestinal cancer	30.9 (168)	38.1 (80)	22.5 (69)	33.0 (92)	1 and 3 > 2
Gynecological cancer	16.9 (92)	18.1 (38)	20.6 (63)	14.3 (40)	NS
Lung cancer	12.7 (69)	9.5 (20)	11.1 (34)	12.2 (34)	NS
Prior cancer treatment					Χ^2^ =14.48, *p* = .106
No prior treatment	24.4 (128)	27.8 (57)	22.2 (66)	27.0 (74)	
Only surgery, CTX, or RT	41.3 (217)	39.5 (81)	45.1 (134)	42.0 (115)	
Surgery and CTX, or surgery and	21.1 (111)	23.4 (48)	20.2 (60)	14.2 (39)	
RT, or CTX and RT					
Surgery and CTX and RT	13.1 (69)	9.3 (19)	12.5 (37)	16.8 (46)	
Metastatic sites					Χ^2^ = 16.36, *p* = .060
No metastasis	31.7 (169)	29.0 (60)	32.9 (100)	35.5 (98)	
Only lymph node metastasis	17.8 (95)	27.1 (56)	25.3 (77)	22.8 (63)	
Only metastatic disease in other sites	23.3 (124)	22.2 (46)	20.1 (61)	17.4 (48)	
Metastatic disease in lymph nodes and other sites	27.2 (145)	21.7 (45)	21.7 (66)	24.3 (67)	
Receipt of targeted therapy (% yes)	34.6 (182)	24.4 (50)	29.7 (90)	25.3 (70)	Χ^2^ = 11.35, *p* = .010 0 > 1 and 4
CTX regimen					Χ^2^ = 19.95, *p* = .003
Only CTX	65.4 (344)	75.6 (155)	70.3 (213)	74.7 (207)	0 < 1 and 3
Only targeted therapy	4.9 (26)	0.5 (1)	2.6 (8)	1.4 (4)	0 > 1
Both CTX and targeted therapy	29.7 (156)	23.9 (49)	27.1 (82)	66 (23.8)	NS
Cycle length					KW = 19.54, *p* < .001
14 day cycle	37.8 (204)	48.8 (101)	36.5 (111)	51.6 (142)	0 and 2 > 3;
21 day cycle	53.5 (289)	45.4 (94)	57.2 (174)	41.5 (114)	0 > 1
28 day cycle	8.7 (47)	5.8 (12)	6.3 (19)	6.9 (19)	
Emetogenicity of the CTX regimen					KW = 22.89, *p* < .001 0 < 1, 2 and 3
Minimal/low	24.4 (132)	12.6 (26)	16.8 (51)	18.2 (50)	
Moderate	61.0 (330)	68.6 (142)	61.8 (188)	54.5 (150)	
High	14.6 (79)	18.8 (39)	21.4 (65)	27.3 (75)	
Antiemetic regimen					Χ^2^ = 34.49, *p* < .001
None	10.1 (53)	3.0 (6)	5.3 (16)	6.4 (17)	0 > 1
Steroid alone or serotonin receptor antagonist alone	23.4 (123)	22.2 (45)	18.3 (55)	15.8 (42)	NS
Serotonin receptor antagonist and steroid	46.8 (246)	51.2 (104)	49.8 (150)	44.4 (118)	NS
NK-1 receptor antagonist and two other antiemetics	19.8 (104)	23.6 (48)	26.6 (80)	33.5 (89)	0 < 3

^a^Total number of metastatic sites evaluated was 9

^+^Reference group

*CIN* chemotherapy-induced nausea; *CTX* chemotherapy; *kg* kilograms; *KW* Kruskal Wallis; *m*^2^ meters squared; pw = pairwise; *NK-1* neurokinin-1; *NS* not significant, *RT* radiation therapy, *SD* standard deviation

#### Neuropsychological symptom scores

Compared to the Both Low-PMF and Changing CIN-Low PMF classes, the other two classes reported higher levels of depression, trait anxiety, sleep disturbance, morning and evening fatigue, and pain interference, as well as lower levels of cognitive function and evening energy (Table 7). Compared to the Both Low-PMF class, the Changing CIN-High PMF and Both High-PMF classes reported lower levels of morning energy, were more likely to report cancer and/or non-cancer pain, and a higher number of pain locations. Compared to the other three classes, the Both High-PMF class had higher state anxiety scores.

**Table 7:** Differences in Neuropsychological Symptom Severity Scores among the CIN and Evening Fatigue Latent Profiles at Enrollment

Neuropsychological Symptom Scores*	No CIN and Low Evening Fatigue	Changing CIN and Low Evening Fatigue	Changing CIN and High Evening Fatigue	High CIN and High Evening Fatigue	Statistics
	(0)	(1)	(2)	(3)	
	40.6% (*n*=543)	15.7% (*n*=210)	22.9% (*n*=306)	20.9% (*n*=279)	
	Mean (SD)	Mean (SD)	Mean (SD)	Mean (SD)	
Center for Epidemiological Studies Depression Scale (≥ 16.0)	10.3 (8.5)	10.9 (7.9)	14.8 (10.4)	17.1 (10.4)	F = 38.96, *p* < .001 0 and 1 < 2 and 3; 2 < 3
Trait Anxiety Inventory (≥ 32.2)	32.9 (9.7)	33.6 (10.0)	36.9 (10.8)	38.7 (10.7)	F = 23.14, *p* < .001 0 and 1 < 2 and 3
State Anxiety Inventory ≥ 31.8)	31.2 (11.2)	32.5 (11.5)	35.4 (13.2)	38.4 (12.8)	F = 24.16, *p* < .001 0, 1 and 2 < 3; 0 < 2
Attentional Function Index (< 5 low, 5–7.5 moderate, > 7.5 high)	6.8 (1.7)	6.9 (1.8)	6.0 (1.7)	5.7 (1.8)	F = 31.66, *p* < .001 0 and 1 > 2 and 3
General Sleep Disturbance Scale (≥ 43.0)	46.7 (19.5)	46.5 (18.1)	56.8 (18.3)	63.3 (19.6)	F = 56.44, *p* < .001 0 and 1 < 2 and 3; 2 < 3
Morning fatigue (≥ 3.2)	2.5 (2.1)	2.2 (1.9)	3.8 (2.2)	4.2 (2.3)	F = 59.21, *p* < .001 0 and 1 < 2 and 3
Evening fatigue (≥ 5.6)	4.8 (2.2)	3.5 (1.7)	6.4 (1.6)	6.5 (1.6)	F = 147.26, *p* < .001 0 and 1 < 2 and 3; 0 > 1
Morning energy (≤ 6.2)	4.6 (2.3)	4.5 (2.4)	4.2 (2.1)	4.1 (2.1)	F = 5.24, *p* = .001 0 > 2 and 3
Evening energy (≤ 3.5)	3.8 (2.0)	3.9 (2.0)	3.2 (2.0)	3.2 (1.9)	F = 10.50, *p* < .001 0 and 1 > 2 and 3
Type of pain (% (*n*))					Χ^2^ = 46.54, *p* < .001
No pain	33.8 (181)	28.3 (58)	19.9 (59)	22.2 (61)	0 > 2 and 3
Only non-cancer pain	18.1 (97)	17.6 (36)	13.1 (39)	13.5 (37)	NS
Only cancer pain	22.0 (118)	25.4 (52)	36.0 (107)	25.1 (69)	0 and 3 < 2
Both cancer and non-cancer pain	26.1 (140)	28.8 (59)	31.0 (92)	39.3 (108)	0 < 3
Worst pain intensity	5.8 (2.5)	5.4 (2.7)	6.1 (2.4)	6.8 (2.4)	F = 8.27, *p* < .001 0, 1 and 2 < 3
Number of pain locations (out of 45)	26.6 (22.1)	29.4 (21.5)	33.5 (19.6)	32.4 (20.2)	F = 8.81, *p* < .001 0 < 2 and 3
Pain interference	2.6 (2.4)	2.4 (2.1)	3.2 (2.5)	4.3 (2.5)	F = 24.88, *p* < .001 0 and 1 < 2 and 3; 2 < 3

*Numbers in parentheses represent clinically meaningful cutpoint scores for the symptom measures

*CIN* chemotherapy-induced nausea; *SD* standard deviation; *NS* not significant

#### Gastrointestinal symptom occurrence rates

Compared to the Both Low-PMF class, the Changing CIN-High PMF class and the Both High-PMF classes reported higher occurrence rates for dry mouth, feeling bloated, and abdominal cramps (Table 8). Compared to the Both Low-PMF class, the other three classes reported higher occurrence rates for vomiting, diarrhea, lack of appetite, constipation, and change in the way food tastes.

**Table 8:** Differences in the occurrence of gastrointestinal symptoms among the CIN and evening fatigue latent profiles at enrollment

Occurrence of symptoms	No CIN and Low Evening Fatigue (0) 40.6% (*n* = 543)	Changing CIN and Low Evening Fatigue (1) 15.7% (*n* = 210)	Changing CIN and High Evening Fatigue (2) 22.9% (*n* = 306)	High CIN and High Evening Fatigue (3) 20.9% (*n* = 279)	Statistics
	% (*n*)	% (*n*)	% (*n*)	% (*n*)	
Dry mouth	38.6 (209)	41.8 (87)	50.7 (154)	55.6 (153)	*Χ*^2^ = 26.32, *p* <.001 0 < 2 and 3; 1 < 3
Feeling bloated	23.8 (129)	30.3 (63)	37.8 (115)	48.4 (133)	*Χ*^2^ = 53.91, *p* <.001 0 < 2 and 3 1 < 3
Vomiting	3.5 (19)	20.2 (42)	13.2 (40)	22.9 (63)	*Χ*^2^ = 79.55, *p* <.001 0 < 1, 2, and 3; 2 < 3
Diarrhea	21.4 (116)	37.0 (77)	33.9 (103)	35.3 (97)	*Χ*^2^ = 29.91, *p* <.001 0 < 1, 2 and 3
Lack of appetite	28.8 (156)	42.8 (89)	43.8 (133)	62.2 (171)	*Χ*^2^ = 85.43, *p* <.001 0 < 1, 2 and 3; 1 and 2 < 3
Increased appetite	22.1 (120)	26.0 (54)	28.3 (86)	30.5 (84)	*Χ*^2^ = 7.99, *p* =.046 No significant pairwise contrasts
Abdominal cramps	15.9 (86)	21.2 (44)	24.7 (75)	34.2 (94)	*Χ*^2^ = 36.24, *p* <.001 0 < 2 and 3; 1 < 3
Difficulty swallowing	8.3 (45)	16.3 (34)	12.8 (39)	23.6 (65)	*Χ*^2^ = 37.58, *p* <.001 0 < 1 and 3; 2 < 3
Mouth sores	17.0 (92)	17.3 (36)	22.0 (67)	30.2 (83)	*Χ*^2^ = 21.23, *p* <.001 0 and 1 < 3
Weight loss	20.1 (109)	25.5 (53)	27.3 (83)	32.7 (90)	*Χ*^2^ = 16.43, *p* <.001 0 < 3
Weight gain	24.0 (130)	27.4 (57)	25.3 (77)	26.5 (73)	*Χ*^2^ = 1.21, *p* =.752
Constipation	33.8 (183)	45.7 (95)	48.4 (147)	55.6 (153)	*Χ*^2^ = 40.70, *p* <.001 0 < 1, 2 and 3
Change in way food tastes	39.5 (214)	51.0 (106)	54.3 (165)	62.2 (171)	*Χ*^2^ = 42.39, *p* <.001 0 < 1, 2 and 3

*CIN* chemotherapy-induced nausea

## Discussion

This study is the first to use LCPA to identify subgroups of oncology patients with distinct joint profiles for CIN with either morning fatigue or evening fatigue over two cycles of chemotherapy. Across both LCPAs, 60% of the sample reported CIN with occurrence rates that ranged from approximately 30% to 90%. In addition, wide variations were found in both morning and evening fatigue severity scores depending on the distinct profile. These initial findings suggest that CIN co-occurs with both morning and evening fatigue. Future studies need to determine which symptom is driving the occurrence/severity of the other symptom.

While it is not readily apparent why five distinct profiles were found with the morning analyses and four with the evening analyses (Figs. 1 and 2), some patterns are worth noting. For both LCPA that included patients without CIN (i.e., 40.6% of the sample), morning and evening fatigue scores were below the clinically meaningful cutoffs. Equally important, for both LCPAs, the worse class had high levels of both symptoms and constituted between 8.1% (morning) and 20.9% (evening) of the sample. A final pattern that is worth noting is that for one or two of the morning and evening profiles, the occurrence rates for CIN changed with the highest rates reported in the weeks after the administration of chemotherapy (i.e., weeks 2 and 5). These findings suggest that within the various patient profiles, while both morning and evening fatigue exhibit relatively stable trajectories, nausea is a much more dynamic symptom.

Additional research is warranted to explain these findings including an evaluation of diurnal variations in the occurrence of nausea and their association with morning and evening fatigue; the influence of adherence with and patterns of use of anti-emetic regimens on latent class membership; and associations between the use of pharmacologic and non-pharmacologic interventions to manage both morning and evening fatigue and latent class membership. Equally important, as noted in the “Introduction” section, CIN and fatigue share common underlying mechanisms (e.g., inflammatory processes [20], alterations in gut-brain axis bidirectional signaling [20, 29]). While studied with individual symptoms, future research needs to evaluate the mechanisms that underlie the co-occurrence of the two symptoms, as well as the inter-individual differences in the severity of the combined symptom profiles. The remainder of the discussion focuses on the common risk factors for the worse co-occurrence profiles across the two LCPAs.

### Common risk factors for worse CIN and fatigue profiles

#### Demographic and clinical factors

Table 9 provides a summary of the characteristics associated with membership in the worse joint profiles compared to the Both Low-AMF and Both Low-PMF classes. Common demographic and clinical characteristics associated with the worse CIN AND fatigue profiles (i.e., High CIN-Moderate AMF, Both High-AMF, and Both High-PMF) were younger age, lower annual income, lower functional status, higher likelihood of reporting depression, and receipt of a neurokinin-1 receptor antagonist and two other antiemetics. Consistent with previous reports, younger age was associated with the occurrence of CIN [46] and higher fatigue severity [47, 48]. This association may be explained by age-related changes in inflammatory processes [49, 50] and/or a “response shift” in older patients’ perceptions of their symptom experience [51]. One potential explanation for the associations with lower annual income is that cancer treatment may impose a significant financial burden on patients (i.e., increased financial toxicity [52]) that decreases their ability to pay for pharmacologic (e.g., antiemetics) and non-pharmacologic (i.e., physical therapy) interventions.

**Table 9:** Characteristics associated with membership in the Joint CIN-Morning Fatigue and Joint CIN-Evening Fatigue classes compared to the No CIN and Low Fatigue classes

Characteristics•	CIN-Morning Fatigue classes^+^				CIN-Evening Fatigue classes^a^		
	1	2	3	4	1	2	3
Demographic characteristics							
Younger age	■	■	■	■	■	■	■
More likely to be female		■	■			■	
Less likely to be married or partnered				■			
More likely to live alone				■			
More likely to have a lower annual income			■	■	■		■
More likely to have childcare responsibilities			■			■	■
Less likely to exercise on a regular basis				■			
Less likely to report a current or past history of smoking					■		
Clinical characteristics							
Lower KPS score		■	■	■		■	■
Higher number of comorbidities				■			■
Higher SCQ score				■			■
Higher MAX2 score		■				■	
More likely to report kidney disease		■		■			
More likely to report anemia or blood disease				■		■	
More likely to report depression		■	■	■		■	■
More likely to report back pain				■			
Less likely to be diagnosed with gastrointestinal cancer		■					
Less likely to receive targeted therapy			■		■		■
More likely to receive only chemotherapy			■		■		■
Less likely to receive only targeted therapy	■				■		
More likely to receive a 14-day cycle of chemotherapy					■		■
More likely to receive highly emetogenic chemotherapy	■	■	■		■	■	■
More likely to receive some type of antiemetics	■				■		
More likely to receive serotonin receptor antagonist and steroid	■						
More likely to receive NK-1 RA and two other antiemetics		■	■	■			■
Neuropsychological symptoms							
Higher depression		■	■	■		■	■
Higher trait anxiety			■	■		■	■
Higher state anxiety			■	■		■	■
Lower attentional function			■	■		■	■
Higher sleep disturbance		■	■	■		■	■
Higher morning fatigue		■	■	■		■	■
Higher evening fatigue		■	■	■		■	■
Lower morning energy			■	■		■	■
Lower evening energy				■		■	■
Type of pain Less likely to have no pain Less likely to have only non-cancer pain More likely to have only cancer pain More likely to have both cancer and non-cancer pain		■ ■	■ ■	■ ■		■ ■	■ ■
Higher worst pain intensity				■			■
Higher number of pain locations			■	■		■	■
Higher pain interference			■	■		■	■
Gastrointestinal symptoms							
Dry mouth			■	■		■	■
Feeling bloated			■	■		■	■
Vomiting	■	■	■	■	■	■	■
Diarrhea	■		■	■	■	■	■
Lack of appetite	■		■	■	■	■	■
Abdominal cramps			■	■		■	■
Difficulty swallowing			■	■	■		■
Mouth sores			■				■
Weight loss			■	■			■
Constipation	■		■	■	■	■	■
Change in way food tastes			■	■	■	■	■

*CIN* chemotherapy-induced nausea, *KPS* Karnofsky Performance Status score, *NK-1* neurokinin-1, *RA* receptor antagonist, *SCQ* Self-administered comorbidity questionnaire

•Comparisons done with the No CIN and Low Fatigue classes

^+^ CIN-Morning Fatigue classes: 1 = Changing CIN and Low Morning Fatigue class; 2 = Changing CIN and Changing Morning Fatigue class; 3 = High CIN and Moderate Morning Fatigue class; 4 = High CIN and High Morning Fatigue class

■Indicates the presence of the risk factor compared with the No CIN and Low Fatigue classes

^a^CIN-Evening Fatigue classes: 1 = Changing CIN and Low Evening Fatigue class; Changing CIN and High Evening Fatigue class; 3 = High CIN and High Evening Fatigue class

Consistent with previous reports from our group [8, 12] and others [53], lower physical function was associated with the worse profiles for both LCPAs. In terms of KPS scores, compared to the Both Low-AMF or Both Low-PMF classes, differences in these scores for the High CIN-Moderate AMF class (Cohen’s *d* = 0.6), Both High-AMF class (Cohen’s *d* = 1.0), and Both High-PMF class (Cohen’s *d* = 0.7) represent clinically meaningful decrements in functional status. Given the established evidence that regular exercise can decrease fatigue [54], as well as recent evidence that suggests that physical activity is associated with reductions in nausea and vomiting in patients with lung cancer [55], patients warrant referrals to physical therapy for an exercise prescription and ongoing assessments of their adherence with the regimen and its efficacy.

#### Neuropsychological symptoms

Compared to the Both Low-AMF or Both Low-PMF classes, patients in the two worse profiles for both LCPAs reported higher scores for depression, state and trait anxiety, sleep disturbance, cognitive impairment, and pain interference, as well as lower scores for morning and evening energy. In addition, the scores reported by the patients in these profiles represent clinically meaningful levels of each of these symptoms (see Tables 4 and 7).

While no studies evaluated symptom burden in patients with distinct CIN and morning or evening fatigue profiles, our findings are consistent with previous reports that demonstrated associations between more severe CIN and higher levels of depression [5, 7], anxiety [5, 7], sleep disturbance [5, 7, 56], and pain [5]. Similarly, positive associations were found between average fatigue scores and higher levels of depression [5, 7], anxiety [5, 7], sleep disturbance [5, 7], cognitive impairment [57], and pain [5]. It is interesting to note that no studies were identified that documented an association between CIN and cognitive impairment. However, in the current study, the AFI scores for the worse classes suggest moderate decrements in cognitive function.

In terms of specific symptoms, among the two worse profiles for the morning and evening LCPAs, 31.8% and 34.3%, respectively, met the criteria for subsyndromal depression (i.e., CES-D scores of between 8 and 15) [36]. In addition, within these two classes, 55.7% of the patients in the morning and 42.5% in the evening LCPAs had CES-D scores that warrant clinical evaluation for depression. These percentages are consistent with previously reported depression rates of 17% to 45% in patients with cancer [58]. Previous studies noted that depression is a risk factor for both CIN and fatigue [3, 5, 59]. Common underlying mechanisms for these three symptoms (e.g., systemic inflammation, disruption of the hypothalamic-pituitary-adrenal axis and/or dysregulation of neurotransmitters including serotonin and substance P [60–64]) may explain these inter-relationships. Future studies need to evaluate the longitudinal relationships among these three symptoms.

Findings from a systematic review determined that the global prevalence rate of anxiety in patients with cancer is 31% [65]. In the current study, except for the Changing CIN-Low AMF class, trait anxiety scores were above the clinically meaningful cutoff for all of the other profiles. In many studies, a trait anxiety score of ≥ 40 indicates a need for a clinical evaluation for an anxiety disorder [37]. Across the two worst morning and evening LCPAs, 51.2% and 37.2% of the patients, respectively, met this cutoff score. While anxiety or “psychological distress” is common in patients receiving chemotherapy, more studies have evaluated for associations between nausea and anxiety compared to nausea and fatigue. In fact, higher levels of anxiety are associated with anticipatory and persistent nausea in patients receiving chemotherapy [66]. Findings from the current study suggest that anxiety is exacerbated by the co-occurrence of more severe CIN and fatigue. While the molecular mechanisms that underlie anxiety are not well understood, findings from a systematic review suggest that alterations in the HPA axis, serotonergic signaling, and gamma-aminobutyric acid metabolism contribute to the occurrence and severity of this symptom [67].

Across all of the profiles in the morning and evening LCPAs, sleep disturbance scores were above the clinically meaningful cutoff. While previous joint LPAs of sleep disturbance and morning and evening fatigue are not reported, the higher sleep disturbance scores in the worse profiles make clinical sense and are supported by cross-sectional analyses of patients receiving chemotherapy [68–70]. In addition, studies documented positive relationships between the severity of nausea and sleep disturbance in oncology patients [6, 56]. A future analysis will evaluate for distinct patient profiles using longitudinal measures of CIN and sleep disturbance.

The AFI evaluates patients’ perceived effectiveness in performing daily activities that are supported by attention and working memory [71]. Across the two worst morning and evening LCAs, AFI scores suggest moderate to high levels of cognitive impairment. In our previous studies with the same sample [72, 73], a joint LPA of AFI and evening fatigue scores demonstrated a dose response relationship between the two symptoms with patients classified into Low, Moderate, and High classes. No studies were identified that evaluated the relationships between nausea and cognitive impairment in patients receiving chemotherapy. Given that higher levels of stress were associated with membership in the Moderate and High joint fatigue and cognitive function profiles [73] and stress contributes to increases in CIN [11], a joint LCPA of CIN and AFI scores is warranted.

Previous work from our group [16] and others [74] demonstrated that decrements in energy are a distinct symptom from fatigue. Energy is described as an individual’s potential to perform physical and mental activities [74]. While joint latent variable models of fatigue and energy or CIN and energy are not available, for the two worst morning and evening LCPAs, both the morning and evening energy scores were below the clinically meaningful cutoffs. Of note, the morning energy levels for all of these patients were extremely low. Additional research is warranted to determine the specific causes for these significant decrements in energy.

The relationships between the various pain characteristics (i.e., type of pain, worst pain intensity, number of pain locations, pain interference) and the various morning and evening CIN and fatigue profiles are complex (Tables 4 and 7). In our previous joint LPA of morning fatigue and pain with the same sample [75], five distinct profiles were identified, with 44% of the sample having high levels of both morning fatigue and pain. Other cross-sectional studies of patients with cancer support this positive association [76, 77]. Less is known about the relationship between CIN and pain in patients receiving chemotherapy and warrants evaluation in future studies.

#### Gastrointestinal symptoms

The current study is the first to evaluate for associations between distinct joint profiles for CIN with either morning fatigue or evening fatigue and occurrence rates for common gastrointestinal symptoms in patients receiving chemotherapy. As shown in Table 9, the two worse profiles for the morning and evening LCPAs had the highest occurrence rates for the majority of the gastrointestinal symptoms. These findings are consistent with studies that identified associations between CIN and other GI symptoms using a variety of analytic techniques [78–80]. In a study of patients with breast cancer receiving chemotherapy [78], using network analysis, a gastrointestinal symptom cluster was identified that included nausea as the sentinel symptom and lack of appetite and vomiting. In a study of patients with lung cancer [79], using network analysis, a digestive symptom cluster was identified that included nausea, poor appetite, constipation, vomiting, and weight loss. However, no sentinel symptom was identified for this cluster. In another study of patients with ovarian cancer [80], in the multivariable analysis, nausea was associated with abdominal bloating, bowel disturbances, lack of appetite, vomiting, and weight loss. While the list of gastrointestinal symptoms in the current study was more comprehensive, these findings, taken together, suggest that multiple gastrointestinal symptoms co-occur at higher rates in patients with more severe CIN and fatigue profiles.

As noted in the “Introduction” section, in our previous analyses with the same sample [20, 28, 29], several common pathways involved in inflammation (e.g., cytokine-cytokine receptor interaction, chemokine signaling) and alterations in the gut-brain axis (e.g., intestinal immune network for IgA) were perturbed for CIN, as well as for morning and evening fatigue (Table 1). In addition, as noted throughout the “Discussion” section, many of these mechanisms are associated with other common symptoms (e.g., depression, anxiety) in patients receiving chemotherapy. An overall evaluation of the symptom burden of patients in the worse profiles for the morning and evening LCPAs suggests that the multiple mechanisms that underlie these symptoms may have additive or synergistic effects in some patients. Future studies need to determine the sentinel symptom that occurs as a result of these interaction effects; develop targeted interventions for this sentinel symptom; and determine the effects of various pharmacologic and non-pharmacologic interventions on reducing symptom burden among patients with the worse profiles.

## Limitations

Several limitations warrant consideration. Given that the sample was primarily female, white, and well-educated, our findings may not generalize to more diverse samples. Since patients had received at least one cycle of chemotherapy, future research needs to evaluate symptoms prior to the initiation of chemotherapy, as well as changes in the joint CIN and fatigue profiles over the entire course of chemotherapy and into survivorship. Given that data were not collected on doses of antiemetics prescribed and changes in antiemetic prescriptions, patients’ adherence with their antiemetic regimen was not evaluated and may have contributed to the changing CIN profiles. In addition, a history of motion sickness as a potential risk factor for CIN was not evaluated [81, 82].

## Implications for practice and research

Being the first study to identify joint profiles for CIN and morning and evening fatigue, these findings require replication. In addition, research is needed on the molecular mechanisms that underlie the co-occurrence of these two common symptoms between and among the distinct profiles. Based on the risk factors listed in Table 9, clinicians need to be alert for patients with a high comorbidity burden and a poorer functional status. Oncology clinicians need to consult with primary care providers to ensure the effective management of patients’ chronic conditions. Referrals are warranted to physical therapy to improve patients’ functional status. Equally important, given the significant symptom burden in the worse profiles, clinicians need to perform comprehensive symptom assessments with all patients receiving chemotherapy and initiate interventions. Clinicians need to evaluate if patients are taking their anti-emetics as prescribed and initiate changes to the prescription if needed. Given the higher prevalence rates for the majority of the gastrointestinal symptoms, patients need to be evaluated for nutritional deficits. Clinicians need to assess hydration status and caloric intake; the need for vitamins and mineral supplements; and whether a referral to a dietician is warranted.

## Data Availability

Data are available from the corresponding author following the completion of a data sharing agreement with the University of California, San Francisco.

## References

[1] Miaskowski C, Cooper BA, Melisko M, Chen LM, Mastick J, West C et al (2014) Disease and treatment characteristics do not predict symptom occurrence profiles in oncology outpatients receiving chemotherapy. Cancer 120(15):2371–2378. 10.1002/cncr.28699 24797450 10.1002/cncr.28699 PMC4108553

[2] National Comprehensive Cancer Network: Antiemetics. http://www.nccn.org/professionals/physician_gls/pdf/antiemesis.pdf (2024). Accessed.

[3] Ma Y, He B, Jiang M, Yang Y, Wang C, Huang C et al (2020) Prevalence and risk factors of cancer-related fatigue: a systematic review and meta-analysis. Int J Nurs Stud 111:103707. 10.1016/j.ijnurstu.2020.103707 32920423 10.1016/j.ijnurstu.2020.103707

[4] Thong MSY, van Noorden CJF, Steindorf K, Arndt V (2020) Cancer-related fatigue: causes and current treatment options. Curr Treat Options Oncol 21(2):17. 10.1007/s11864-020-0707-5 32025928 10.1007/s11864-020-0707-5 PMC8660748

[5] Crane TE, Badger TA, Sikorskii A, Segrin C, Hsu CH, Rosenfeld AG (2020) Symptom profiles of Latina breast cancer survivors: a latent class analysis. Nurs Res 69(4):264–271. 10.1097/NNR.0000000000000434 32604142 10.1097/NNR.0000000000000434

[6] Peoples AR, Roscoe JA, Block RC, Heckler CE, Ryan JL, Mustian KM et al (2017) Nausea and disturbed sleep as predictors of cancer-related fatigue in breast cancer patients: a multicenter NCORP study. Support Care Cancer 25(4):1271–1278. 10.1007/s00520-016-3520-8 27995318 10.1007/s00520-016-3520-8 PMC5323277

[7] Whisenant M, Wong B, Mitchell SA, Beck SL, Mooney K (2019) Symptom trajectories are associated with co-occurring symptoms during chemotherapy for breast cancer. J Pain Symptom Manage 57(2):183–189. 10.1016/j.jpainsymman.2018.11.010 30453052 10.1016/j.jpainsymman.2018.11.010 PMC6348053

[8] Singh K, Pituch K, Zhu Q, Gu H, Ernst B, Tofthagen C et al (2023) Distinct nausea profiles are associated with gastrointestinal symptoms in oncology patients receiving chemotherapy. Cancer Nurs 46(2):92–102. 10.1097/NCC.0000000000001076 35671438 10.1097/NCC.0000000000001076 PMC9437145

[9] Singh K, Kober KM, Paul SM, Hammer M, Wright F, Conley YP et al (2020) Gastrointestinal symptoms are associated with trajectories of chemotherapy-induced nausea. Support Care Cancer 28(5):2205–2215. 10.1007/s00520-019-05031-5 31428931 10.1007/s00520-019-05031-5 PMC7028490

[10] Singh K, Paul SM, Kober KM, Conley YP, Wright F, Levine JD et al (2020) Neuropsychological symptoms and intrusive thoughts are associated with worse trajectories of chemotherapy-induced nausea. J Pain Symptom Manage 59(3):668–678. 10.1016/j.jpainsymman.2019.10.023 31689477 10.1016/j.jpainsymman.2019.10.023 PMC7024637

[11] Singh KP, Cooper BA, Tofthagen CS, Fryer JD, Singh P, Pituch K et al (2023) Higher levels of stress and neuropsychological symptoms are associated with a high nausea profile in patients with cancer receiving chemotherapy. Oncol Nurs Forum 50(4):461–473. 10.1188/23.Onf.461-473 37677748 10.1188/23.ONF.461-473

[12] Wright F, Dunn LB, Paul SM, Conley YP, Levine JD, Hammer MJ et al (2019) Morning fatigue severity profiles in oncology outpatients receiving chemotherapy. Cancer Nurs 42(5):355–364. 10.1097/NCC.0000000000000626 30024437 10.1097/NCC.0000000000000626 PMC6336532

[13] Wright F, D’Eramo Melkus G, Hammer M, Schmidt BL, Knobf MT, Paul SM et al (2015) Predictors and trajectories of morning fatigue are distinct from evening fatigue. J Pain Symptom Manage 50(2):176–189. 10.1016/j.jpainsymman.2015.02.016 25828559 10.1016/j.jpainsymman.2015.02.016 PMC4526314

[14] Wright F, D’Eramo Melkus G, Hammer M, Schmidt BL, Knobf MT, Paul SM et al (2015) Trajectories of evening fatigue in oncology outpatients receiving chemotherapy. J Pain Symptom Manage 50(2):163–175. 10.1016/j.jpainsymman.2015.02.015 25828560 10.1016/j.jpainsymman.2015.02.015 PMC4526403

[15] Wright F, Cooper BA, Conley YP, Hammer MJ, Chen LM, Paul SM et al (2017) Distinct evening fatigue profiles in oncology outpatients receiving chemotherapy. Fatigue : biomedicine, health & behavior 5(3):131–144. 10.1080/21641846.2017.1322233 10.1080/21641846.2017.1322233 PMC5926814 29725554

[16] Lerdal A, Kottorp A, Gay C, Aouizerat BE, Lee KA, Miaskowski C (2016) A Rasch analysis of assessments of morning and evening fatigue in oncology patients using the Lee Fatigue Scale. J Pain Symptom Manage 51(6):1002–1012. 10.1016/j.jpainsymman.2015.12.331 26975624 10.1016/j.jpainsymman.2015.12.331 PMC4902715

[17] Wright F, Cooper BA, Hammer MJ, Paul SM, Conley YP, Levine JD et al (2024) Stress exposures contribute to worse joint morning and evening fatigue profiles in patients with cancer during chemotherapy. Oncol Nurs Forum 51(2):89–106. 10.1188/24.Onf.89-106 38442280 10.1188/24.ONF.89-106

[18] Wright F, Cooper BA, Paul SM, Hammer MJ, Conley YP, Levine JD et al (2023) Distinct profiles of morning and evening fatigue co-occurrence in patients during chemotherapy. Nurs Res 72(4):259–271. 10.1097/nnr.0000000000000661 37084242 10.1097/NNR.0000000000000661 PMC10330127

[19] Wright F, Hammer M, Paul SM, Aouizerat BE, Kober KM, Conley YP et al (2017) Inflammatory pathway genes associated with inter-individual variability in the trajectories of morning and evening fatigue in patients receiving chemotherapy. Cytokine 91:187–210. 10.1016/j.cyto.2016.12.023 28110208 10.1016/j.cyto.2016.12.023 PMC5318191

[20] Kober KM, Harris C, Conley YP, Dhruva A, Dokiparthi V, Hammer MJ et al (2023) Perturbations in common and distinct inflammatory pathways associated with morning and evening fatigue in outpatients receiving chemotherapy. Cancer Med 12(6):7369–7380. 10.1002/cam4.5435 36373573 10.1002/cam4.5435 PMC10067125

[21] Chen W, Zhao Y, Dai Y, Nie K (2022) Gastrointestinal inflammation plays a critical role in chemotherapy-induced nausea and vomiting. Eur J Pharmacol 936:175379. 10.1016/j.ejphar.2022.175379 36356927 10.1016/j.ejphar.2022.175379

[22] Crowder SL, Hoogland AI, Welniak TL, LaFranchise EA, Carpenter KM, Li D et al (2022) Metagenomics and chemotherapy-induced nausea: a roadmap for future research. Cancer 128(3):461–470. 10.1002/cncr.33892 34643945 10.1002/cncr.33892 PMC8776572

[23] Vanrusselt D, Sleurs C, Arif M, Lemiere J, Verschueren S, Uyttebroeck A (2023) Biomarkers of fatigue in oncology: a systematic review. Crit Rev Oncol Hematol 194:104245. 10.1016/j.critrevonc.2023.104245 38141868 10.1016/j.critrevonc.2023.104245

[24] Singh KP, Dhruva AA, Flowers E, Kober KM, Miaskowski C (2018) A review of the literature on the relationships between genetic polymorphisms and chemotherapy-induced nausea and vomiting. Crit Rev Oncol Hematol 121:51–61. 10.1016/j.critrevonc.2017.11.012 29279099 10.1016/j.critrevonc.2017.11.012 PMC5777158

[25] Belloni S, Caruso R, Giacon C, Baroni I, Conte G, Magon A et al (2024) Microbiome-modifiers for cancer-related fatigue management: a systematic review. Semin Oncol Nurs 40(2):151619. 10.1016/j.soncn.2024.151619 38503656 10.1016/j.soncn.2024.151619

[26] König RS, Albrich WC, Kahlert CR, Bahr LS, Löber U, Vernazza P et al (2021) The gut microbiome in myalgic encephalomyelitis (ME)/chronic fatigue syndrome (CFS). Front Immunol 12:628741. 10.3389/fimmu.2021.628741 35046929 10.3389/fimmu.2021.628741 PMC8761622

[27] Raizen DM, Mullington J, Anaclet C, Clarke G, Critchley H, Dantzer R et al (2023) Beyond the symptom: the biology of fatigue. Sleep. 10.1093/sleep/zsad069 37224457 10.1093/sleep/zsad069 PMC10485572

[28] Singh K, Cao H, Miaskowski C, Conley YP, Hammer M, Wright F et al (2021) Perturbations in endocytotic and apoptotic pathways are associated with chemotherapy-induced nausea. Biol Res Nurs 23(2):238–247. 10.1177/1099800420951271 32815385 10.1177/1099800420951271 PMC8822189

[29] Singh KP, Dhruva A, Flowers E, Paul SM, Hammer MJ, Wright F, et al. Alterations in patterns of gene expression and perturbed pathways in the gut-brain axis are associated with chemotherapy-induced nausea. J Pain Symptom Manage. 2020;59(6):1248–59 e5. 10.1016/j.jpainsymman.2019.12.352. 10.1016/j.jpainsymman.2019.12.352 PMC7239734 31923555

[30] Karnofsky D. (1997) Performance scale. Factors that influence the therapeutic response in cancer: a comprehensive treatise. New York: Plenum Press.

[31] Sangha O, Stucki G, Liang MH, Fossel AH, Katz JN (2003) The Self-Administered Comorbidity Questionnaire: a new method to assess comorbidity for clinical and health services research. Arthritis Rheum 49(2):156–163. 10.1002/art.10993 12687505 10.1002/art.10993

[32] Babor TF, Higgins-Biddle JC, Saunders JB, Monteiro MG (2001) AUDIT: The Alcohol Use Disorders Identification Test: guidelines for use in primary care. World Health Organization, Geneva, Switzerland

[33] Portenoy RK, Thaler HT, Kornblith AB, Lepore JM, Friedlander-Klar H, Kiyasu E et al (1994) The Memorial Symptom Assessment Scale: an instrument for the evaluation of symptom prevalence, characteristics and distress. Eur J Cancer. 10.1016/0959-8049(94)90182-1 7999421 10.1016/0959-8049(94)90182-1

[34] Lee KA, Hicks G, Nino-Murcia G (1991) Validity and reliability of a scale to assess fatigue. Psychiatry Res 36(3):291–298. 10.1016/0165-1781(91)90027-m 2062970 10.1016/0165-1781(91)90027-m

[35] Fletcher BS, Paul SM, Dodd MJ, Schumacher K, West C, Cooper B et al (2008) Prevalence, severity, and impact of symptoms on female family caregivers of patients at the initiation of radiation therapy for prostate cancer. J Clin Oncol 26(4):599–605. 10.1200/JCO.2007.12.2838 18235118 10.1200/JCO.2007.12.2838

[36] Radloff LS (1977) The CES-D scale: a self-report depression scale for research in the general population. Appl Psychol Measure 1(3):385–401

[37] Spielberger CG, Gorsuch RL, Suchene R, Vagg PR, Jacobs GA (1983) Manual for the State-Anxiety (Form Y): Self Evaluation Questionnaire. Consulting Psychologists Press, Palo Alto, CA

[38] Cimprich B, So H, Ronis DL, Trask C (2005) Pre-treatment factors related to cognitive functioning in women newly diagnosed with breast cancer. Psychooncology 14(1):70–78. 10.1002/pon.821 15386786 10.1002/pon.821

[39] Daut RL, Cleeland CS, Flanery RC (1983) Development of the Wisconsin brief pain questionnaire to assess pain in cancer and other diseases. Pain 17(2):197–210. 10.1016/0304-3959(83)90143-4 6646795 10.1016/0304-3959(83)90143-4

[40] Vermunt JK, Magidson J (2002) Applied latent class analysis. In: Hagenaara JA, McCutcheon AL (eds) Latent class cluster analysis. Canbridge University Press, pp 89–106

[41] Lanza ST, Flaherty BP, Collins LM. Latent class and latent transition analysis. . In: Schinka JA, Velicer WF, editors. Handbook of psychology: Vol 2 Research methods in psychology. John Wiley and Sons.; 2003. p. 663–85.

[42] Muthen LK, Muthen BO (1998–2020) (Mplus user's guide (8th ed.). 8th ed. Los Angeles, CA: Muthen & Muthen.

[43] Nylund KL, Asparouhov T, Muthén BO (2007) Deciding on the number of classes in latent class analysis and growth mixture modeling: a Monte Carlo simulation study. Struct Equ Modeling 14(4):535–569

[44] Celeux G, Soromenho G (1996) An entropy criterion for assessing the number of clusters in a mixture model. J Classif 13(2):195–212

[45] Muthen B, Shedden K (1999) Finite mixture modeling with mixture outcomes using the EM algorithm. Biometrics 55(2):463–469. 10.1111/j.0006-341x.1999.00463.x 11318201 10.1111/j.0006-341x.1999.00463.x

[46] Iihara H, Fujii H, Yoshimi C, Yamada M, Suzuki A, Matsuhashi N et al (2016) Control of chemotherapy-induced nausea in patients receiving outpatient cancer chemotherapy. Int J Clin Oncol 21(2):409–418. 10.1007/s10147-015-0908-2 26475354 10.1007/s10147-015-0908-2 PMC4824820

[47] Soltow D, Given BA, Given CW (2010) Relationship between age and symptoms of pain and fatigue in adults undergoing treatment for cancer. Cancer Nurs 33:296–303 20467311 10.1097/NCC.0b013e3181ce5a1a

[48] Wada S, Shimizu K, Inoguchi H, Shimoda H, Yoshiuchi K, Akechi T et al (2015) The association between depressive symptoms and age in cancer patients: a multicenter cross-sectional study. J Pain Symptom Manage 50(6):768–777. 10.1016/j.jpainsymman.2015.07.011 26300022 10.1016/j.jpainsymman.2015.07.011

[49] Bower J (2014) Cancer-related fatigue–mechanisms, risk factors, and treatments. Nat Rev Clin Oncol 11(10):597–609 25113839 10.1038/nrclinonc.2014.127 PMC4664449

[50] Bower JE (2019) The role of neuro-immune interactions in cancer-related fatigue: biobehavioral risk factors and mechanisms. Cancer 125(3):353–364. 10.1002/cncr.31790 30602059 10.1002/cncr.31790 PMC6502236

[51] Schwartz C, Sprangers M (1999) Methodological approaches for assessing response shift in longitudinal health-related quality-of-life research. Soc Sci Med 48(11):1531–1548 10400255 10.1016/s0277-9536(99)00047-7

[52] Shankaran V, Jolly S, Blough D, Ramsey S (2012) Risk factors for financial hardship in patients receiving adjuvant chemotherapy for colon cancer: a population-based exploratory analysis. J Clin Oncol 30(14):1608–1614 22412136 10.1200/JCO.2011.37.9511

[53] Minton O, Berger A, Barsevick A, Cramp F, Goedendorp M, Mitchell S et al (2013) Cancer related fatigue and its impact on functioning. Cancer 119:2124–2130 23695924 10.1002/cncr.28058

[54] Fontvieille A, Parent-Roberge H, Fülöp T, Pavic M, Riesco E (2024) The mechanisms underlying the beneficial impact of aerobic training on cancer-related fatigue: a conceptual review. Cancers (Basel). 10.3390/cancers16050990 38473351 10.3390/cancers16050990 PMC10930711

[55] Bai L, Ni L, Lu J, Zhang YY, Yin Y, Zhang W et al (2024) Relationship between nausea and vomiting and physical activity in patients with lung cancer undergoing first chemotherapy. Front Oncol 14:1396637. 10.3389/fonc.2024.1396637 39114312 10.3389/fonc.2024.1396637 PMC11303201

[56] Jung D, Lee KM, Kim WH, Lee JY, Kim TY, Im SA et al (2016) Longitudinal association of poor sleep quality with chemotherapy-induced nausea and vomiting in patients with breast cancer. Psychosom Med 78(8):959–965. 10.1097/psy.0000000000000372 27428859 10.1097/PSY.0000000000000372

[57] Henneghan A (2016) Modifiable factors and cognitive dysfunction in breast cancer survivors: a mixed method systematic review. Support Care Cancer 24(1):481–497 26416490 10.1007/s00520-015-2927-y

[58] Wen S, Xiao H, Yang Y (2019) The risk factors for depression in cancer patients undergoing chemotherapy: a systematic review. Support Care Cancer. 10.1007/s00520-018-4466-9 30225571 10.1007/s00520-018-4466-9

[59] Hockenberry MJ, Hooke MC, Rodgers C, Taylor O, Koerner KM, Mitby P et al (2017) Symptom trajectories in children receiving treatment for leukemia: a latent class growth analysis with multitrajectory modeling. J Pain Symptom Manage 54(1):1–8. 10.1016/j.jpainsymman.2017.03.002 28433546 10.1016/j.jpainsymman.2017.03.002 PMC6431078

[60] Ullah I, Ayaz M (2023) A re-consideration of neural/receptor mechanisms in chemotherapy-induced nausea and vomiting: current scenario and future perspective. Pharmacol Rep 75(5):1126–1137. 10.1007/s43440-023-00514-z 37584820 10.1007/s43440-023-00514-z

[61] Was H, Borkowska A, Bagues A, Tu L, Liu JYH, Lu Z et al (2022) Mechanisms of chemotherapy-induced neurotoxicity Front Pharmacol 13:750507. 10.3389/fphar.2022.750507 35418856 10.3389/fphar.2022.750507 PMC8996259

[62] Dantzer R, Chelette B, Vichaya EG, West AP, Grossberg A (2025) The metabolic basis of cancer-related fatigue. Neurosci Biobehav Rev 169:106035. 10.1016/j.neubiorev.2025.106035 39892436 10.1016/j.neubiorev.2025.106035 PMC11866516

[63] Kanter NG, Cohen-Woods S, Balfour DA, Burt MG, Waterman AL, Koczwara B (2024) Hypothalamic-pituitary-adrenal axis dysfunction in people with cancer: a systematic review. Cancer Med 13(22):e70366. 10.1002/cam4.70366 39569439 10.1002/cam4.70366 PMC11579619

[64] Chen Y, Lu Y, Chen S, Liu P, He J, Jiang L et al (2025) Molecular mechanisms and clinical value of the correlation between depression and cancer. Med Oncol 42(6):214. 10.1007/s12032-025-02763-9 40381122 10.1007/s12032-025-02763-9

[65] Getie A, Ayalneh M, Bimerew M (2025) Global prevalence and determinant factors of pain, depression, and anxiety among cancer patients: an umbrella review of systematic reviews and meta-analyses. BMC Psychiatry 25(1):156. 10.1186/s12888-025-06599-5 39972435 10.1186/s12888-025-06599-5 PMC11841195

[66] Mosa ASM, Hossain AM, Lavoie BJ, Yoo I (2020) Patient-related risk factors for chemotherapy-induced nausea and vomiting: a systematic review. Front Pharmacol 11:329. 10.3389/fphar.2020.00329 32296333 10.3389/fphar.2020.00329 PMC7138899

[67] Merkouris E, Brasinika A, Patsiavoura M, Siniosoglou C, Tsiptsios D, Triantafyllis AS et al (2025) Molecular basis of anxiety: a comprehensive review of 2014-2024 clinical and preclinical studies. Int J Mol Sci. 10.3390/ijms26115417 40508224 10.3390/ijms26115417 PMC12155146

[68] Ee C, Kay S, Reynolds A, Lovato N, Lacey J, Koczwara B (2024) Lifestyle and integrative oncology interventions for cancer-related fatigue and sleep disturbances. Maturitas 187:108056. 10.1016/j.maturitas.2024.108056 38981156 10.1016/j.maturitas.2024.108056

[69] Greeley KM, Rash J, Tulk J, Savard J, Seal M, Urquhart R et al (2025) Impact and mechanisms of cognitive behavioral therapy for insomnia on fatigue among cancer survivors: a secondary analysis of a randomized controlled trial. Sleep. 10.1093/sleep/zsaf014 39826090 10.1093/sleep/zsaf014 PMC12163127

[70] Hendy A, Ibrahim RK, Darwish A, Al Sabbah S, Shalby AYM, Khubrani R et al (2025) Sleep disturbance, cancer-related fatigue, and depression as determinants of quality of life among breast cancer patients undergoing chemotherapy: a cross-sectional study. BMC Cancer 25(1):1122. 10.1186/s12885-025-14538-6 40597987 10.1186/s12885-025-14538-6 PMC12211318

[71] Cimprich B, Visovatti M, Ronis DL (2011) The attentional function index--a self-report cognitive measure. Psychooncology 20(2):194–202. 10.1002/pon.1729 20213858 10.1002/pon.1729

[72] Morse L, Kober KM, Viele C, Cooper BA, Paul SM, Conley YP et al (2021) Subgroups of patients undergoing chemotherapy with distinct cognitive fatigue and evening physical fatigue profiles. Support Care Cancer 29(12):7985–7998. 10.1007/s00520-021-06410-7 34218321 10.1007/s00520-021-06410-7

[73] Morse L, Paul SM, Cooper BA, Oppegaard K, Shin J, Calvo-Schimmel A et al (2023) Higher stress in oncology patients is associated with cognitive and evening physical fatigue severity. J Pain Symptom Manage 65(3):203–215. 10.1016/j.jpainsymman.2022.11.017 36423801 10.1016/j.jpainsymman.2022.11.017 PMC11189665

[74] Lerdal A (2002) A theoretical extension of the concept of energy through an empirical study. Scand J Caring Sci 16(2):197–206. 10.1046/j.1471-6712.2002.00079.x 12000674 10.1046/j.1471-6712.2002.00079.x

[75] Bouvron B, Mackin L, Kober KM, Paul SM, Cooper BA, Conley YP et al (2022) Impact of worst pain severity and morning fatigue profiles on oncology outpatients’ symptom burden and quality of life. Support Care Cancer 30(12):9929–9944. 10.1007/s00520-022-07431-6 36355215 10.1007/s00520-022-07431-6

[76] Liu D, Weng JS, Ke X, Wu XY, Huang ST (2023) The relationship between cancer-related fatigue, quality of life and pain among cancer patients. Int J Nurs Sci 10(1):111–116. 10.1016/j.ijnss.2022.12.006 36860712 10.1016/j.ijnss.2022.12.006 PMC9969059

[77] Romero SAD, Jones L, Bauml JM, Li QS, Cohen RB, Mao JJ (2018) The association between fatigue and pain symptoms and decreased physical activity after cancer. Support Care Cancer 26(10):3423–3430. 10.1007/s00520-018-4203-4 29675547 10.1007/s00520-018-4203-4 PMC6117206

[78] Liang M, Zhong T, Knobf MT, Chen L, Xu M, Cheng B et al (2024) Sentinel and networked symptoms in patients with breast cancer undergoing chemotherapy. Eur J Oncol Nurs 70:102566. 10.1016/j.ejon.2024.102566 38513452 10.1016/j.ejon.2024.102566

[79] Luo Y, Mao D, Zhang L, Yang Z, Miao J, Zhang L (2024) Identification of symptom clusters and sentinel symptoms during the first cycle of chemotherapy in patients with lung cancer. Support Care Cancer 32(6):385. 10.1007/s00520-024-08600-5 38801450 10.1007/s00520-024-08600-5 PMC11130015

[80] Donovan HS, Hagan TL, Campbell GB, Boisen MM, Rosenblum LM, Edwards RP et al (2016) Nausea as a sentinel symptom for cytotoxic chemotherapy effects on the gut-brain axis among women receiving treatment for recurrent ovarian cancer: an exploratory analysis. Support Care Cancer 24(6):2635–2642. 10.1007/s00520-015-3071-4 26746209 10.1007/s00520-015-3071-4 PMC4846512

[81] Naito Y, Kai Y, Ishikawa T, Fujita T, Uehara K, Doihara H et al (2020) Chemotherapy-induced nausea and vomiting in patients with breast cancer: a prospective cohort study. Breast Cancer 27(1):122–128. 10.1007/s12282-019-01001-1 31407150 10.1007/s12282-019-01001-1 PMC6954145

[82] Clemons M, Bouganim N, Smith S, Mazzarello S, Vandermeer L, Segal R et al (2016) Risk model-guided antiemetic prophylaxis vs physician’s choice in patients receiving chemotherapy for early-stage breast cancer: a randomized clinical trial. JAMA Oncol 2(2):225–231. 10.1001/jamaoncol.2015.3730 26562292 10.1001/jamaoncol.2015.3730

